# Two Escape Mechanisms of Influenza A Virus to a Broadly Neutralizing Stalk-Binding Antibody

**DOI:** 10.1371/journal.ppat.1005702

**Published:** 2016-06-28

**Authors:** Ning Chai, Lee R. Swem, Mike Reichelt, Haiyin Chen-Harris, Elizabeth Luis, Summer Park, Ashley Fouts, Patrick Lupardus, Thomas D. Wu, Olga Li, Jacqueline McBride, Michael Lawrence, Min Xu, Man-Wah Tan

**Affiliations:** 1 Infectious Diseases Department, Genentech, South San Francisco, California, United States of America; 2 Pathology Department, Genentech, South San Francisco, California, United States of America; 3 Bioinformatics & Computational Biology Department, Genentech, South San Francisco, California, United States of America; 4 Cancer Immunology Department, Genentech, South San Francisco, California, United States of America; 5 Protein Chemistry Department, Genentech, South San Francisco, California, United States of America; 6 Translational Immunology Department, Genentech, South San Francisco, California, United States of America; 7 Structural Biology Department, Genentech, South San Francisco, California, United States of America; 8 Development Sciences Department, Genentech, South San Francisco, California, United States of America; Icahn School of Medicine at Mount Sinai, UNITED STATES

## Abstract

Broadly neutralizing antibodies targeting the stalk region of influenza A virus (IAV) hemagglutinin (HA) are effective in blocking virus infection both in vitro and in vivo. The highly conserved epitopes recognized by these antibodies are critical for the membrane fusion function of HA and therefore less likely to be permissive for virus mutational escape. Here we report three resistant viruses of the A/Perth/16/2009 strain that were selected in the presence of a broadly neutralizing stalk-binding antibody. The three resistant viruses harbor three different mutations in the HA stalk: (1) Gln387Lys; (2) Asp391Tyr; (3) Asp391Gly. The Gln387Lys mutation completely abolishes binding of the antibody to the HA stalk epitope. The other two mutations, Asp391Tyr and Asp391Gly, do not affect antibody binding at neutral pH and only slightly reduce binding at low pH. Interestingly, they enhance the fusion ability of the HA, representing a novel mechanism that allows productive membrane fusion even in the presence of antibody and hence virus escape from antibody neutralization. Therefore, these mutations illustrate two different resistance mechanisms used by IAV to escape broadly neutralizing stalk-binding antibodies. Compared to the wild type virus, the resistant viruses release fewer progeny viral particles during replication and are more sensitive to Tamiflu, suggesting reduced viral fitness.

## Author Summary

IAV causes seasonal epidemics and periodic pandemics that result in significant morbidity and mortality worldwide. The effectiveness of influenza vaccines is highly variable because the virus evolves rapidly and causes antibody mismatch. The use of neuraminidase inhibitors, the current standard of treatment for IAV infection, is limited by their lack of efficacy beyond 48 hours of symptom onset and by the emergence of drug resistant viruses. Recently, broadly neutralizing antibodies targeting the conserved stalk region of IAV HA have been discovered. These antibodies are able to block the infection of many or even all IAV strains, and hold great promise as the next generation of anti-flu treatment. Nonetheless, virus resistance to these antibodies has not been thoroughly studied despite the common view that broadly neutralizing stalk-binding antibodies are less permissive for mutational escape due to the functional importance of their highly conserved epitopes. In this study, we isolated three resistant viruses to a stalk-binding antibody that was previously shown to neutralize all IAV tested. Interestingly, they use two distinct mechanisms to escape the antibody, abolishing antibody binding or enhancing membrane fusion. Our study emphasizes the need to consider novel escape mechanisms when studying virus resistance to broadly neutralizing stalk-binding antibodies.

## Introduction

Each year influenza virus causes 3 to 5 million cases of severe illness and around half million deaths worldwide [1], with more than 200,000 hospitalizations and approximately 36,000 deaths in the United States alone [2,3]. Beyond causing seasonal flu and epidemics, influenza A virus (IAV) has the potential to generate large pandemics and kill millions of people [4]. Although influenza vaccines are available, they typically elicit strain-specific antibody responses and are thus ineffective against serologically distinct new viral variants. This is exemplified by the mismatch between the 2014 vaccine and the actual H3N2 IAV strain circulating during the 2014/15 winter season [5]. The current standards of treatment for influenza A infection are neuraminidase inhibitors such as oseltamivir phosphate (Tamiflu) and zanamivir (Relenza) that block the function of the viral neuraminidase (NA) protein, thereby blocking efficient viral release from infected cells. Other antiviral drugs such as amantadine, an inhibitor of the viral ion channel M2, have also been used. While these small-molecule inhibitors are effective against susceptible strains, high resistance frequency limits their clinical use. [6,7]. Antiviral resistance and vaccine mismatch can be attributed to the highly error-prone nature of the viral RNA-dependent RNA polymerase, which constantly introduces polymorphisms to viral proteins [8].

Hemagglutinin (HA) is the major surface protein of IAV and the immuno-dominant target of host antibodies. There are currently 18 serologically different HA subtypes (H1–H18) of IAV that are divided into two phylogenetic groups: group 1 that includes H1, H2 and H5, and group 2 that includes H3 and H7 [9]. The subtypes associated with human seasonal and pandemic disease are limited to H1, H2, and H3 while viruses containing H5 and H7 cause sporadic human outbreaks with no sustained human-to-human transmission. HA is synthesized as a precursor polypeptide HA0, which is cleaved by host proteases to yield two subunits, HA1 and HA2. HA1 forms the globular head domain that contains the receptor binding site; HA2 contributes to the stalk region that carries out the membrane fusion function of HA [7]. Because of its accessibility, the head domain contains epitopes for most neutralizing antibodies elicited by virus infection or vaccination, with the majority of them targeting the receptor binding site and surrounding residues [8,10]. However, due to the high sequence variability of the head domain, these antibodies are strain-specific and resistant mutants are readily selected [11,12]. Although the receptor-binding pocket itself is highly conserved, the surrounding residues are variable and contribute to escape from antibody neutralization [13]. There exists broadly neutralizing antibodies (bnAbs) that target the head epitopes [9], but they too display a restricted neutralization spectrum and escape mutants can be generated quickly via in vitro passage under selective pressure [14–17].

In the past few years, bnAbs targeting the conserved HA stalk region have been discovered for IAV [9,13]. These include group 1 antibodies [18–20], group 2 antibodies [21–23], pan-IAV antibodies [24,25] and pan-flu A and B antibodies [26]. Because the epitopes of stalk-binding antibodies are necessary to maintain proper HA trimerization and undergo dramatic yet controlled conformational changes during the fusion process, they are intrinsically less permissive for mutational escape. [13]. Indeed, efforts to select escape mutants for some of these antibodies have been unsuccessful [19,24]. Nevertheless, resistant variants have been isolated for group 1 antibodies C179 [20] and CR6261 [27], and for group 2 antibodies CR8020 [21], CR8043 [22], 042-100809-2F04, 045-051310-2B06 and S6-B01 [23]. While most of the mutations completely abolished antibody binding, a CR6261 escape mutation (His111Leu in HA2) and an S6-B01 escape mutation (Ile384Thr) only partially reduced binding, suggesting an alternative escape mechanism [23,27].

Here we report the elucidation of two mechanisms by which IAV can escape broadly neutralizing stalk-binding antibodies by characterizing three escape mutants to the pan-IAV antibody, 39.29 [25]. One of the mutants contains a mutation in HA that completely abolishes binding to 39.29 as expected. Interestingly, the other two mutants retain significant binding to 39.29 and their ability to escape antibody neutralization correlates with increased membrane fusion ability of the HA, thus representing a novel mechanism to escape the neutralization ability of fusion inhibiting stalk-binding antibodies. Importantly, all these escape mutants show reduced viral fitness, suggesting that they are not fully pathogenic.

## Results

### Selection of viruses resistant to monoclonal antibody (mAb) 39.29

**Fig 1:**
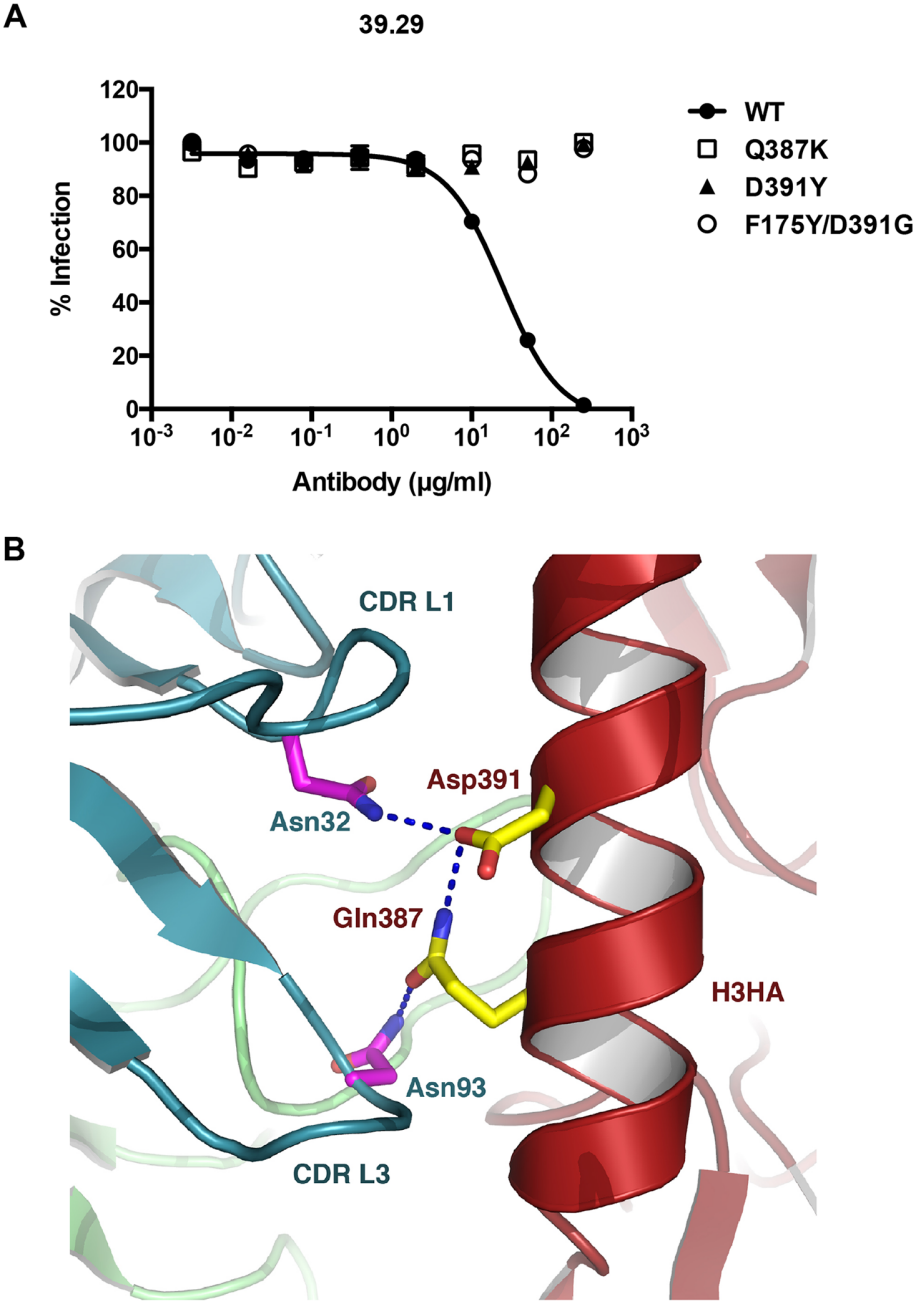
Viruses resistant to mAb 39.29. (A) Micro-neutralization assay was performed on MDCK cells in 96-well plates. WT and mutant A/Perth/16/2009 viruses were incubated with serial dilutions of 39.29 ranging from 0.0032 to 250 μg/ml. Cells were incubated with the virus-antibody mixture for 16 hours prior to immuno-staining with anti-IAV NP and Hoechst 33342. The percentages of infected cells for each virus were normalized to the value at the lowest antibody concentration. The assay was done in triplicate with data presented as Mean +/- SEM (standard error of the mean). (B) The structure is generated from PDB ID, 4KVN with PYMOL. The stalk of A/Perth/16/2009 HA is in red, the light chain of 39.29 Fab is in blue, and the heavy chain is in green. Amino acid side chains for Asn32 (in CDR L1) and Asn93 (in CDR L3) of the light chain, and Asp391 and Gln387 of the HA are represented as sticks.

We previously discovered a stalk-binding mAb 39.29 that was able to neutralize all influenza A strains tested [25]. In an effort to select viruses that are resistant to 39.29, we passaged a group 1 virus (A/California/7/2009, H1N1) and a group 2 virus (A/Perth/16/2009, H3N2) (both were vaccine strains at the time of this study) on MDCK cells in the presence of increasing concentration of 39.29 up to 4x IC90 for A/California/7/2009 and 1x IC90 for A/Perth/16/2009, both high antibody concentrations that inhibited most of viral infection and allowed only minimal cytopathic effect in wells containing non-resistant viruses. After 8 rounds of passage, no resistant virus emerged from A/California/7/2009 in the presence of 39.29; by contrast, resistant viruses were readily selected in the presence of a neutralizing antibody against the head group of A/California/7/2009 HA, with a dominant mutation (Gly172Glu) in HA head, consistent with the common view that broadly neutralizing stalk-binding antibodies are less permissive for mutational escape [8,13]. However, for A/Perth/16/2009, we isolated three resistant viruses in the presence of 39.29. All three resistant viruses completely escaped neutralization by 39.29 (Fig 1A). Sequence analysis revealed individual mutations in the HA gene: (Mutant 1) Gln387Lys (Q387K); (Mutant 2) Asp391Tyr (D391Y); (Mutant 3) Phe175Tyr/Asp391Gly (F175Y/D391G).

We previously determined the crystal structure of 39.29 Fab in complex with the A/Perth/16/2009 HA [25]. As shown in Fig 1B, residues Gln387 and Asp391 are located in Helix A of the HA2 stalk region and make contact with 39.29 light-chain CDRL1 residue Asn32 and CDRL3 residue Asn93, respectively. In addition, the side chains of Q387 and D391 form a hydrogen bond even in the presence of the antibody. This interaction is strongly conserved among 50 published HA structures from the Protein Data Bank that we examined. Phe175 is located in the HA1 head region distant from the Helix A and F175Y is likely an inconsequential random mutation as results obtained with the F175Y/D391G double mutant HA are very similar to those with the D391G single mutant HA, and F175Y single mutant HA behaves like the WT HA (S1 Fig).

### Binding of resistant viruses and mutant HAs to 39.29

**Fig 2:**
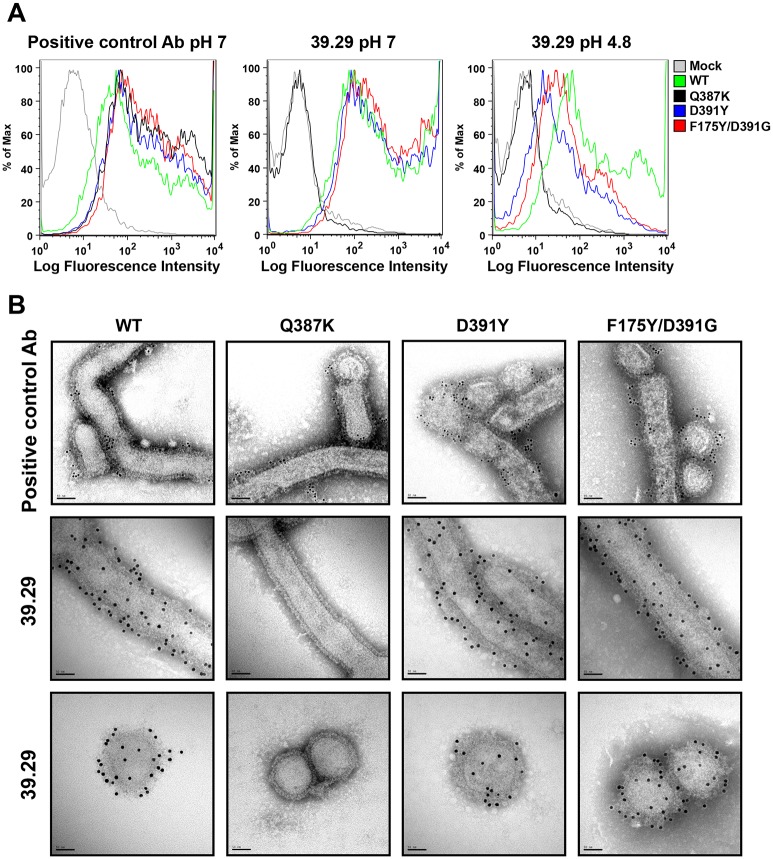
Differential binding properties of the mutants to 39.29. (A) 293T cells expressing the WT or mutant A/Perth/16/2009 HAs were incubated with a positive control antibody (left panel) or 39.29 (middle and right panels) at pH 7 (left and middle panels) or 4.8 (right panel). Flow cytometry profiles are shown. Mock, mock transfected cells. (B) Immunogold EM images of the WT and mutant A/Perth/16/2009 viruses bound by a positive control antibody (top panel with 6 nm gold conjugate) or 39.29 (middle and bottom panels, showing filamentous and spherical particles respectively, with 10 nm gold conjugate). Scale bar is 50 nm. Note the loss of binding of 39.29 to Q387 particles.

A common mechanism by which viruses escape neutralizing antibodies is via mutations that eliminate or severely reduce binding of the antibody. Since all three resistant viruses harbor mutations in the 39.29 epitope, we tested binding of 39.29 to these mutants. We expressed full-length HAs of the wild type (WT) or mutant viruses on the surface of 293T cells and measured antibody binding by flow cytometry and Scatchard assay. As a positive control for protein expression, we used a previously isolated non-neutralizing mAb 36.94 that binds to all IAV HAs tested [25]. All WT and mutant HAs expressed well on cell surface as they all bound 36.94 well (Fig 2A, left panel). As expected, flow cytometry revealed a total loss of binding of the Q387K mutant to 39.29. By contrast, the D391Y and F175Y/D391G mutants bound 39.29 as well as the WT at pH 7 (Fig 2A, middle panel). These observations were confirmed by Scatchard analysis, which measures the equilibrium binding of antibody to the cell surface antigen. WT and the D391Y and F175Y/D391G mutant HAs had similar binding affinity (K_d_) to 39.29 while the Q387K mutant did not bind at all (Table 1).

**Table 1:** Binding affinities between antibodies and HAs at pH7 or pH4.8.

	Positive control Ab		39.29	
HA	pH 7	pH 4.8	pH 7	pH 4.8
**WT**	**2.5**	**23**	**9.3**	**65**
**Q387K**	**4.0**	**22**	**No measurable binding**	**No measurable binding**
**D391Y**	**4.1**	**38**	**11.2**	**165**
**F175Y/D391G**	**4.2**	**20**	**10.7**	**119**

The affinities were determined by the Scatchard equilibrium binding assay. Numbers in table are K_d_ in nM.

One possible scenario for the observed resistance of the D391Y and F175Y/D391G mutants is that although 39.29 binds these mutants at neutral pH, it falls off at low pH in the endosome. To address this, we performed the binding assays at pH 4.8, the average pH of lysosomes [28]. As expected, 39.29 still did not bind the Q387K mutant HA at lower pH. The D391Y and F175Y/D391G mutant HAs showed reduced binding compared to WT, but were still clearly bound by 39.29 as compared to the mock- and Q387K-transfected cells (Fig 2A, right panel). Scatchard analysis confirmed that the Q387K mutation abolished binding of the HA by 39.29 at pH 4.8, whereas HAs harboring the D391Y and F175Y/D391G mutations showed only 2–3 fold reduced affinities to 39.29 compared to WT at this pH (Table 1).

To further confirm the observations on the differential binding of 39.29 to mutant HAs on the surface of 293T cells, we tested the binding of antibodies to WT and mutant virus particles by immunogold staining and EM. Both spherical and filamentous particles were present and bound similarly to the antibodies. Consistent with earlier results, all viruses were bound by 36.94 (Fig 2B, top panel). The WT, D391Y and F175Y/D391G virus particles were bound by 39.29, whereas the Q387K virus particle showed no binding (Fig 2B, middle and bottom panels).

### A novel virus escape mechanism to stalk-binding mAb 39.29

**Fig 3:**
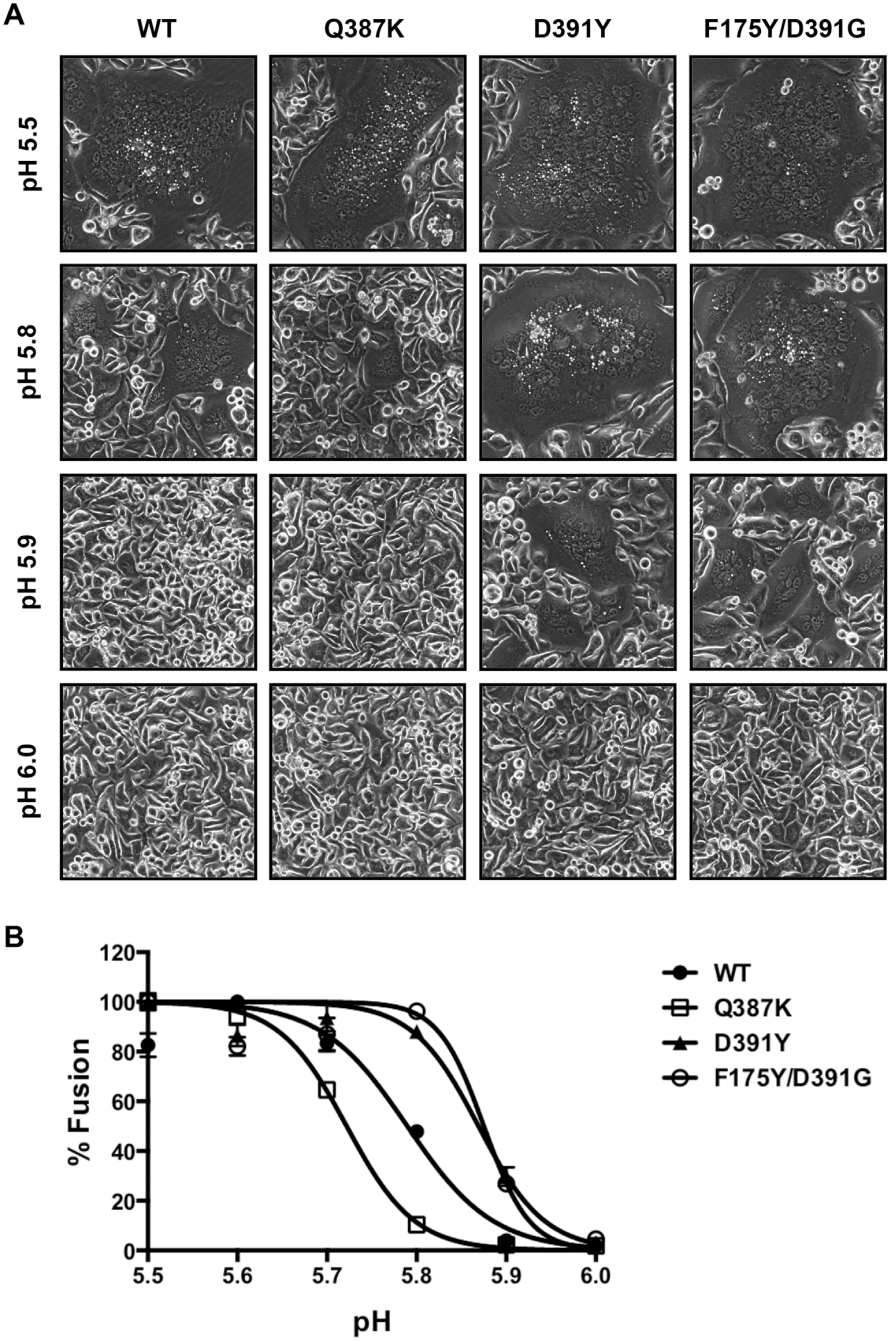
Differential membrane fusion properties of WT and mutant HAs. (A) Hela cells expressing the WT or mutant A/Perth/16/2009 HAs were treated with trypsin to activate HA0 and then incubated with buffers at different pHs for 2 minutes to induce cell-cell fusion. After overnight culture, representative images were obtained under a phase contrast microscope. Note the dramatic difference between the WT and mutant HAs at pH 5.8 and 5.9. (B) Hela cells expressing the WT or mutant A/Perth/16/2009 HAs plus a tetracycline (Tet)-inducible luciferase protein were mixed with Hela Tet-On 3G cells expressing the WT or mutant HAs. Fusion was induced as in (A). After overnight culture, cells were lysed and incubated with a luminescent substrate of the luciferase. Luminescence signals were measured and normalized to the value at pH 5.5 for each HA. The percentages of fusion were plotted at various pH values and the data were fit with a nonlinear regression dose response curve. The assay was done in triplicate with data presented as Mean +/- SEM.

Results from the binding studies suggested that the Q387K mutant escapes 39.29 neutralization by abolishing antibody binding. By contrast, the D391Y and F175Y/D391G mutations allowed viruses to escape 39.29-mediated neutralization despite being bound by the antibody even at low pH. To elucidate the mechanism of resistance by these mutations, and because these mutations are within Helix A, which carries out the important function of membrane fusion, we first examined the impact of these mutations on HA-mediated fusion activity using a well-established cell-cell fusion assay [29]. Hela cells transfected with plasmid encoding the WT or mutant HA were subjected to citric acid/sodium citrate buffer solutions at different pHs and syncytium formation was monitored. Under this buffer system, maximal fusion was achieved at pH 5.5. At pHs between 4.8 and 5.5, no difference in fusion was observed between the WT and mutant HAs (S7 Fig). By contrast, at pH ranging from 5.5–6.0, syncytium formation became pH-dependent for all four HAs, with large syncytia formed at pH 5.5 whereas no syncytia present when pH was raised to 6.0 (Fig 3A). Interestingly, fusion abilities of the WT and mutant HAs could be separated within this pH range. At pH 5.8, D391Y and F175Y/D391G HAs were still able to form large syncytia whereas the syncytia formed by WT HA were much smaller in size. At pH 5.9, D391Y and F175Y/D391G still induced syncytium formation while WT did not. (Fig 3A). By contrast, syncytium formation by Q387K HA was similar to that mediated by WT HA across the range of pHs tested, with the exception of pH 5.8, where the syncytia formed by Q387K HA appeared to be smaller than those induced by WT (Fig 3A).

To quantify the fusion results, we developed a luciferase based cell-cell fusion assay in which Hela cells carrying a luciferase gene under the control of a Tet-inducible promoter were mixed with Hela cells expressing the Tet transactivator protein. The cells were co-transfected with HA-encoding plasmids to allow fusion at low pH, resulting in luciferase expression that was quantified by luminescence. Cells co-transfected with the empty plasmid (no HA) were used as control and gave ignorable background luminescence signals compared to cells expressing HA (S2 Fig). Fusion curves were generated for cells expressing the WT and mutant HAs and they matched well with microscopic results, with biggest separation seen at pH 5.8 (Fig 3B). The curves for D391Y and F175Y/D391G HAs were right-shifted compared to WT HA, quantitatively demonstrating the ability of the D391 mutants to caused membrane fusion at higher pH compared to WT. For example, the pH to achieve 90% fusion was 5.69 for WT, 5.79 for D391Y and 5.82 for F175Y/D391G.

**Fig 4:**
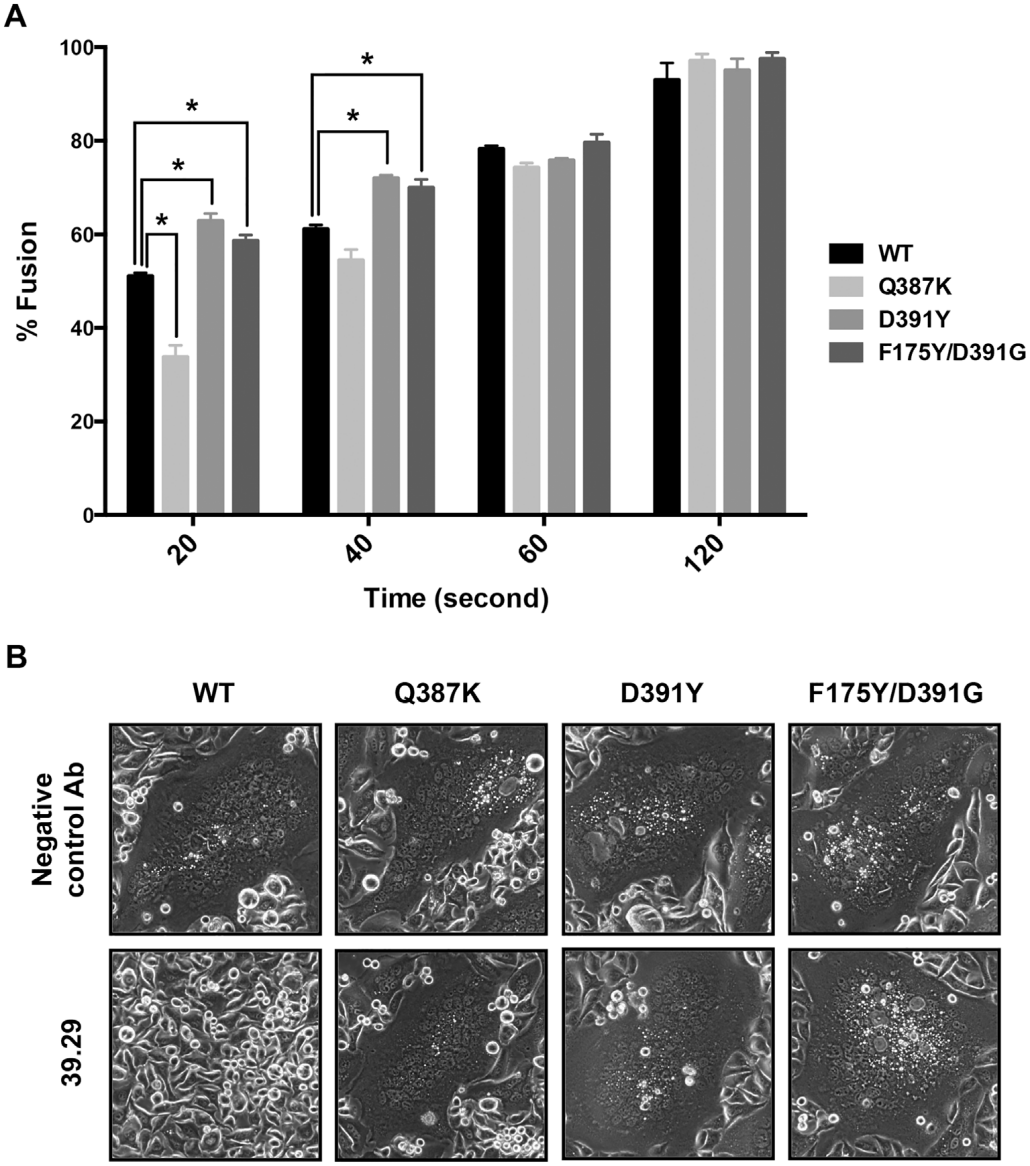
Fusion kinetics of WT and mutant HAs and sensitivity to 39.29. (A) Hela cells expressing the WT or mutant A/Perth/16/2009 HAs plus a tetracycline (Tet)-inducible luciferase protein were mixed with Hela Tet-On 3G cells expressing the WT or mutant HAs. Cells were treated with trypsin to activate HA0 and then incubated with a buffer of pH 5.7 for 20, 40, 60 or 120 seconds to induce cell-cell fusion. After overnight culture, cells were lysed and incubated with a luminescent substrate of the luciferase. Luminescence signals were measured and normalized to the largest value at 120 second for each HA. The percentages of fusion are shown as histograms at each time point. The assay was done in triplicate with data presented as Mean +/- SEM. Statistics were calculated between WT and each of the mutants using a multiple t test with the GraphPad Prism v.6.0 software (* P ≤ 0.05, indicating significant difference). (B) Hela cells expressing the WT or mutant A/Perth/16/2009 HAs were treated with trypsin to activate HA0 and then incubated with either 39.29 or a negative control antibody before pH drop to 5.5 to induce cell-cell fusion. After overnight culture, representative images were obtained under a phase contrast microscope. 39.29 was able to block the fusion mediated by the WT HA but not the mutant HAs.

We next examined the fusion kinetics of WT and mutant HAs at pH 5.7, where WT and mutants were all capable of fusion, over a 2 minute time course. Minimal difference of fusion was observed between WT and mutant HAs when cells were exposed to the low-pH buffer for 60 seconds or longer (Fig 4A). Differences in the rate of fusion became apparent at 40 and 20 second exposures, with the efficiency of fusion: D391Y = F175Y/D391G > WT > Q387K. Statistically, significant difference was observed between WT and each of the three mutant HAs at 20 second, and between WT and D391Y or F175Y/D391G at 40 second. These results are in line with recent studies that the time required to achieve hemifusion of an H3N2 virus is 30–40 seconds [30,31] and demonstrate that D391Y and F175Y/D391G mutants mediate fusion faster than WT, while Q387K is slower. Thus, The D391 mutations apparently both increase the rate of fusion and allow fusion to occur at higher pH.

**Fig 5:**
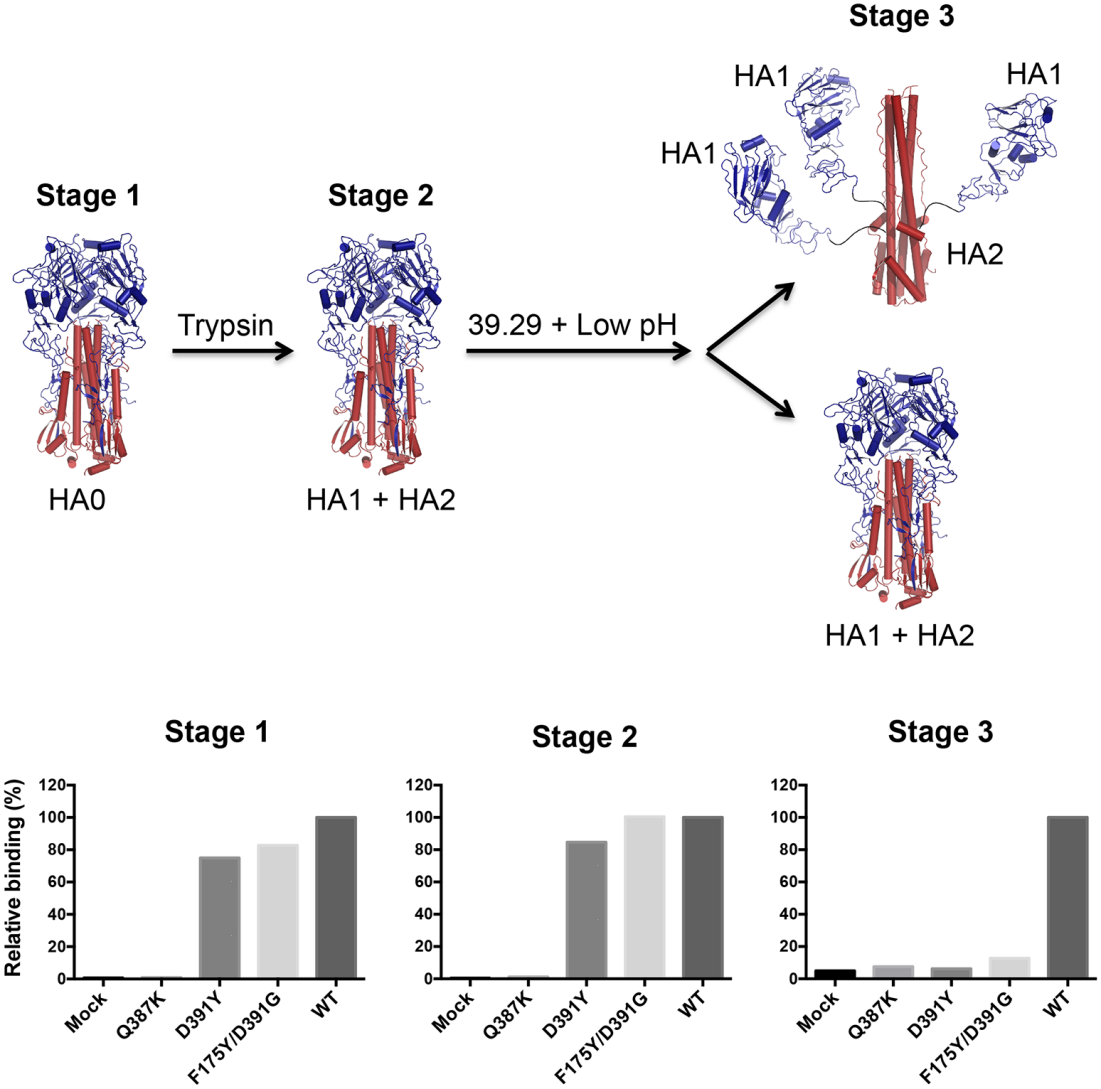
39.39 blocks the low-pH induced conformational change of the WT HA but not the mutant HAs. 293T cells expressing the Q387K, D391Y, F175Y/D391G or WT A/Perth/16/2009 HA were collected before (Stage 1) or after (Stage 2) trypsin treatment to activate HA0. A fraction of trypsin-treated cells were incubated with 39.29 and then subjected to a pH4.8 buffer to induce HA conformational change (Stage 3). Cells at each stage were tested for 39.29 binding by flow cytometry. The mean fluorescence intensities were normalized to the WT and the percentages of binding are shown as histograms. The possible HA conformations at each stage are depicted above the binding data. HA1 is in blue and HA2 is in red. Mock, mock transfected cells.

Since stalk-binding antibodies block the membrane fusion step [30,31], we tested whether the mutants with elevated fusion ability could overcome the block by 39.29 in a fusion assay performed at pH 5.5 when maximal fusion occurred in the absence of antibody. As expected, 39.29 completely blocked the membrane fusion ability of WT HA. By contrast, consistent with its inability to bind the Q387K HA, 39.29 did not block membrane fusion induced by the Q387K mutant (Fig 4B). Interestingly, the D391Y and F175Y/D391G mutants were able to induce giant syncytia in the presence of 39.29 as well as a negative control antibody (Fig 4B). Thus, the enhanced rate of membrane fusion and the ability to cause fusion at higher pH rendered by these mutations appeared to be sufficient for these mutant HAs to overcome the fusion blocking ability of 39.29, which may already have been attenuated by the marginally decreased affinity of the mAb to the mutant HAs at low pH. To confirm these results, we tested whether 39.29 selectively blocks the low pH induced conformational change of the WT HA but not the mutant HAs in an established HA conformational change assay by flow cytometry [21]. As shown in Fig 5, 39.29 did not bind Q387K HA0 whereas it bound D391Y, F175Y/D391G and WT HA0s well (Stage 1). Trypsin cleavage was complete (S3 Fig) but did not alter the binding profile (Fig 5 Stage 2). However, a 2-minute pH drop abolished D391Y and F175Y/D391G binding even with pre-bound 39.29 (Fig 5 Stage 3), indicating that the D391 mutations could overcome the block of HA conformational change by 39.29, possibly due to the enhanced fusion ability of the mutant HAs. By contrast, when 39.29 pre-bound the WT HA before the pH shift, it remained bound after the pH was lowered, preventing the low pH induced conformational change in WT HA. These results are in agreement with the results from the fusion assay that 39.29 blocks membrane fusion mediated by the WT HA but not the mutant HAs.

Taken together, our results from the binding and fusion assays suggested the existence of two different virus escape mechanisms to broadly neutralizing stalk-binding antibody: (a) abolishing antibody binding, as exemplified by the Q387K mutant; (b) enhancing the membrane fusion ability of the HA protein, possibly in conjunction with reduced antibody binding at low pH, as exemplified by the D391Y and the F175Y/D391G mutants.

### Phenotypes of the corresponding mutations in an H1 HA

**Fig 6:**
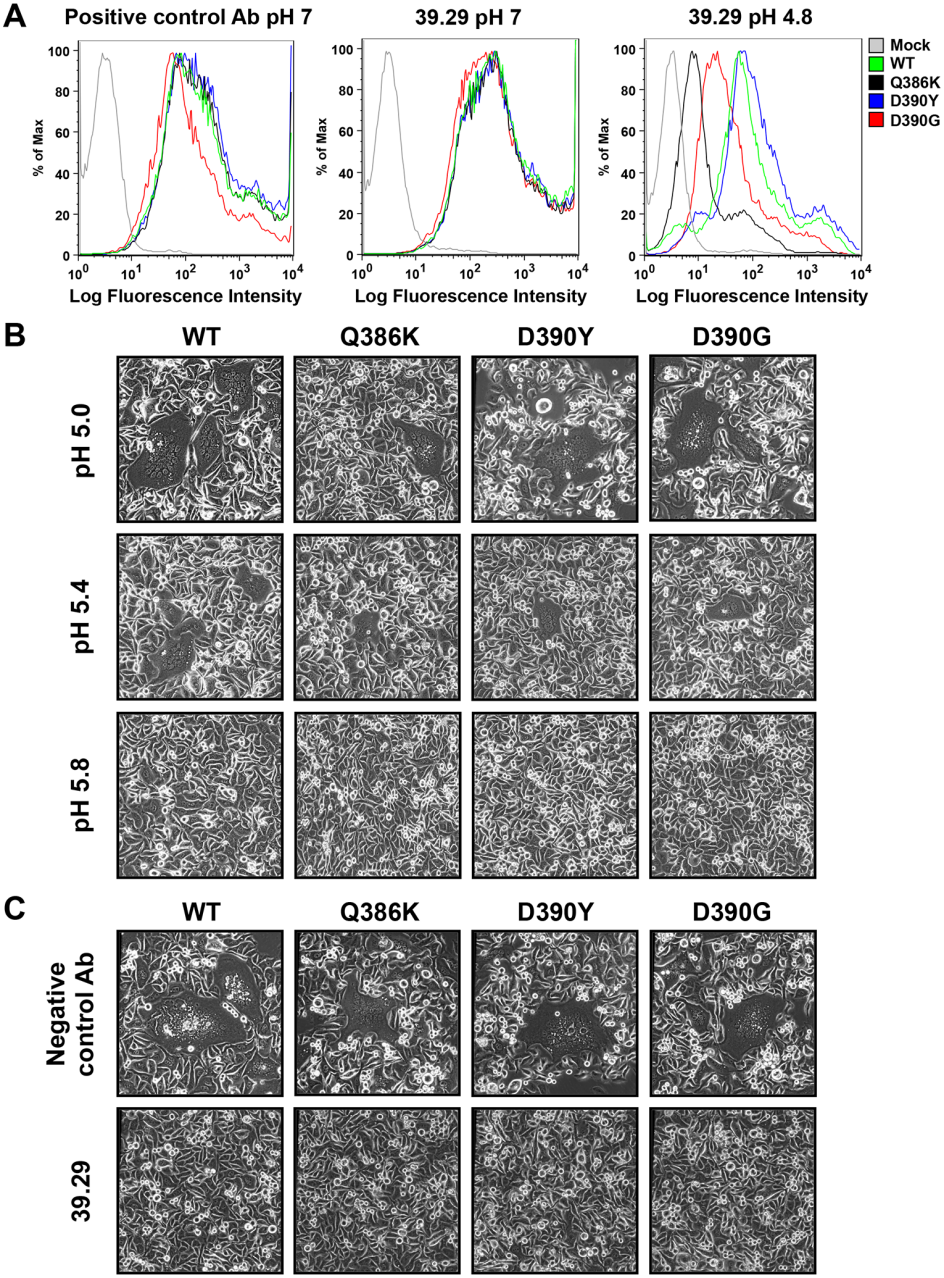
Binding and fusion properties of the Q386K, D390Y and D390G mutant HAs of A/California/7/2009. The Q387K, D391Y and D391G mutations of the A/Perth/16/2009 resistant viruses were introduced into the corresponding residues of the A/California/7/2009 HA to generate the Q386K, D390Y and D390G mutant HAs. (A) 293T cells expressing the WT or mutant A/California/7/2009 HAs were incubated with a positive control antibody (left panel) or 39.29 (middle and right panels) at pH 7 (left and middle panels) or 4.8 (right panel). Flow cytometry profiles are shown. Mock, mock transfected cells. (B) Hela cells expressing the WT or mutant A/California/7/2009 HAs were treated with trypsin to activate HA0 and then incubated with buffers at different pHs for 2 minutes to induce cell-cell fusion. After overnight culture, representative images were obtained under a phase contrast microscope. (C) Hela cells expressing the WT or mutant A/California/7/2009 HAs were treated with trypsin to activate HA0 and then incubated with either 39.29 or a negative control antibody before pH drop to 4.8 to induce cell-cell fusion. After overnight culture, representative images were obtained under a phase contrast microscope. 39.29 was able to block fusion mediated by either the WT or the mutant HAs.

Since no 39.29-resistant virus was isolated from the A/California/7/2009 (H1N1) passage, we introduced the three mutations above into the A/California/7/2009 HA and tested whether they had a different effect on the H1 HA. The three corresponding mutations in H1 HA are Q386K, D390Y and D390G; there is no phenylalanine corresponding to Phe175 in H1 HA. Flow cytometry showed that, unlike the corresponding mutant HAs of the H3 subtype, all three mutant H1 HAs, including Q386K, bound 39.29 as well as the WT at pH 7 (Fig 6A). At pH 4.8, Q386K and D390G showed reduced binding compared to WT; however, they still bound 39.29 significantly (Fig 6A).

We next examined the fusion ability of the WT and mutant H1 HAs in a cell-cell fusion assay on Hela cells. It appears that the A/California/7/2009 HA did not facilitate membrane fusion as well as the A/Perth/16/2009 HA with maximal fusion achieved at pH 5.0 and no syncytia present at pH 5.8 (Fig 6B). The Q386K mutant yielded slightly smaller syncytia compared to the WT HA at all pHs tested, which is consistent with the slightly lower fusion efficiency observed for the Q387K H3 HA mutant. Interestingly, the D390Y or D390G mutations did not enhance fusion at any pH tested (Fig 6B), indicating that structural and functional differences exist in the stalk regions of H1 and H3 HAs despite their highly conserved sequences [30].

### Neither enhanced fusion ability nor reduced antibody binding at low pH is sufficient to escape the fusion block by 39.29

Since the Q386K, D390Y and D390G mutant H1 HAs showed different levels of 39.29 binding at low pH, we tested whether they could escape the fusion block by 39.29 in a fusion assay performed at pH4.8 to ensure maximal fusion in the absence of the antibody. Interestingly, 39.29 completely blocked the fusion mediated by both the WT and the mutant HAs (Fig 6C), including Q386K and D390G that showed significantly reduced binding to 39.29 at pH4.8 (Fig 6A). The inability of these two mutants to escape the fusion block by 39.29 is consistent with their lack of fusion enhancing effect (Fig 6B), and consistent with the fact that no resistant viruses emerged after in vitro passaging of the A/California/7/2009 virus in the presence of 39.29. These results suggest that reduced antibody binding is not sufficient for 39.29 escape; enhanced fusion is likely needed as well.

**Fig 7:**
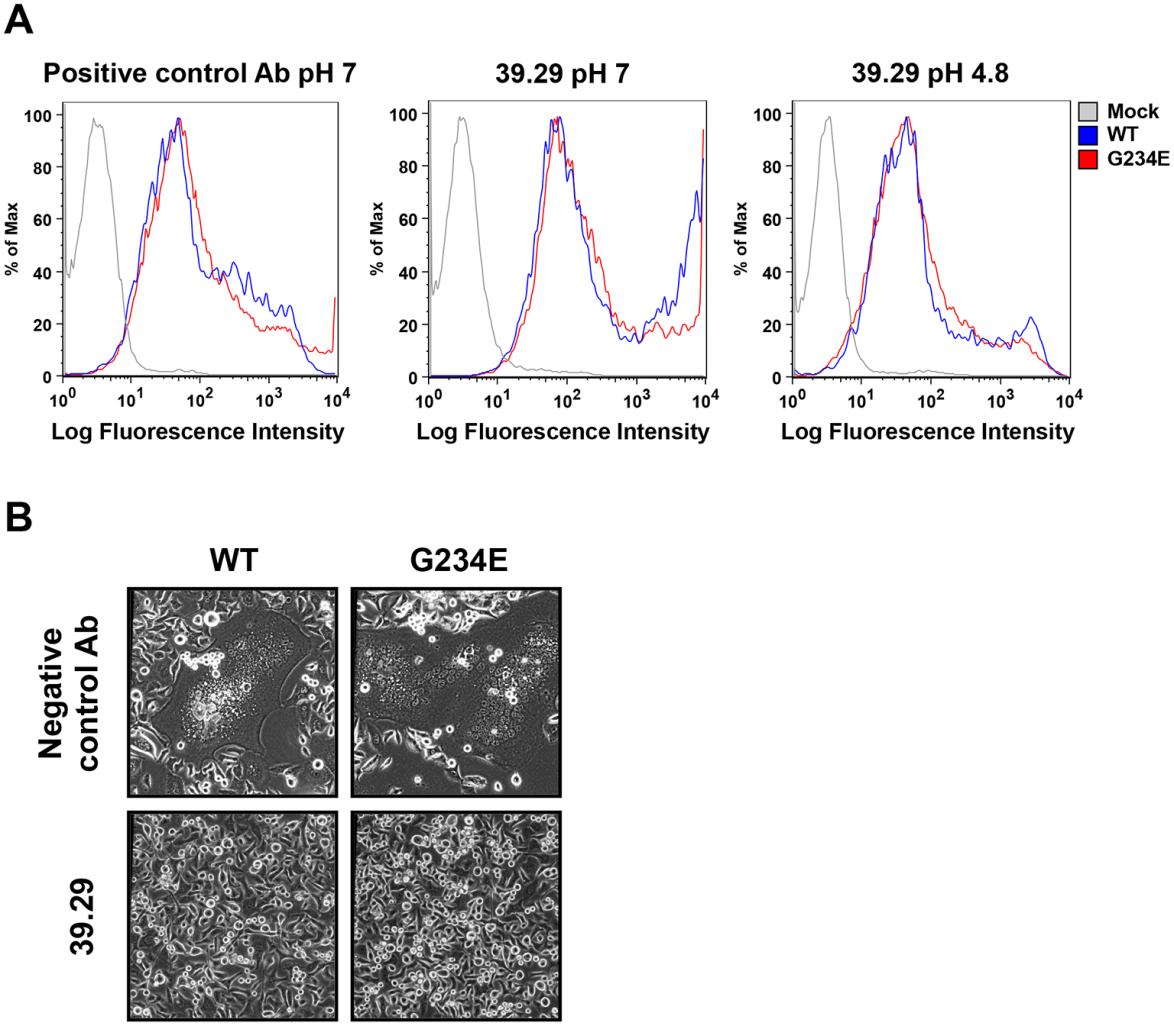
The G234E mutant HA of A/Perth/16/2009 binds and is blocked by 39.29 as well as the WT HA. (A) 293T cells expressing the WT or G234E A/Perth/16/2009 HA were incubated with a positive control antibody (left panel) or 39.29 (middle and right panels) at pH 7 (left and middle panels) or 4.8 (right panel). Flow cytometry profiles are shown. Mock, mock transfected cells. (B) Hela cells expressing the WT or G234E A/Perth/16/2009 HA were treated with trypsin to activate HA0 and then incubated with either 39.29 or a negative control antibody before pH drop to 5.4 to induce maximal cell-cell fusion. After overnight culture, representative images were obtained under a phase contrast microscope. 39.29 was able to block the fusion mediated by the G234E mutant HA.

We next asked whether enhanced fusion is sufficient for 39.29 escape without reduced antibody binding. To test this, we introduced into the A/Perth/16/2009 HA a previously reported fusion-enhancing mutation, G234E, which is distant from the 39.29 epitope [32]. We first confirmed that the G234E mutation indeed enhanced fusion significantly (S4 Fig). Maximal fusion was achieved at pH 5.4 for both WT and G234E HAs. However, at pHs 6.0 and 6.2 when WT HA did not cause any fusion, G234E was still able to induce syncytia. We next showed that G234E HA bound 39.29 as well as the WT at both neutral and low pHs (Fig 7A). Consequently, 39.29 completely blocked the fusion mediated by G234E HA (Fig 7B) despite its enhanced fusion ability. These results suggest that enhanced fusion alone is not sufficient for 39.29 escape.

### All three resistant viruses have growth defects

**Fig 8:**
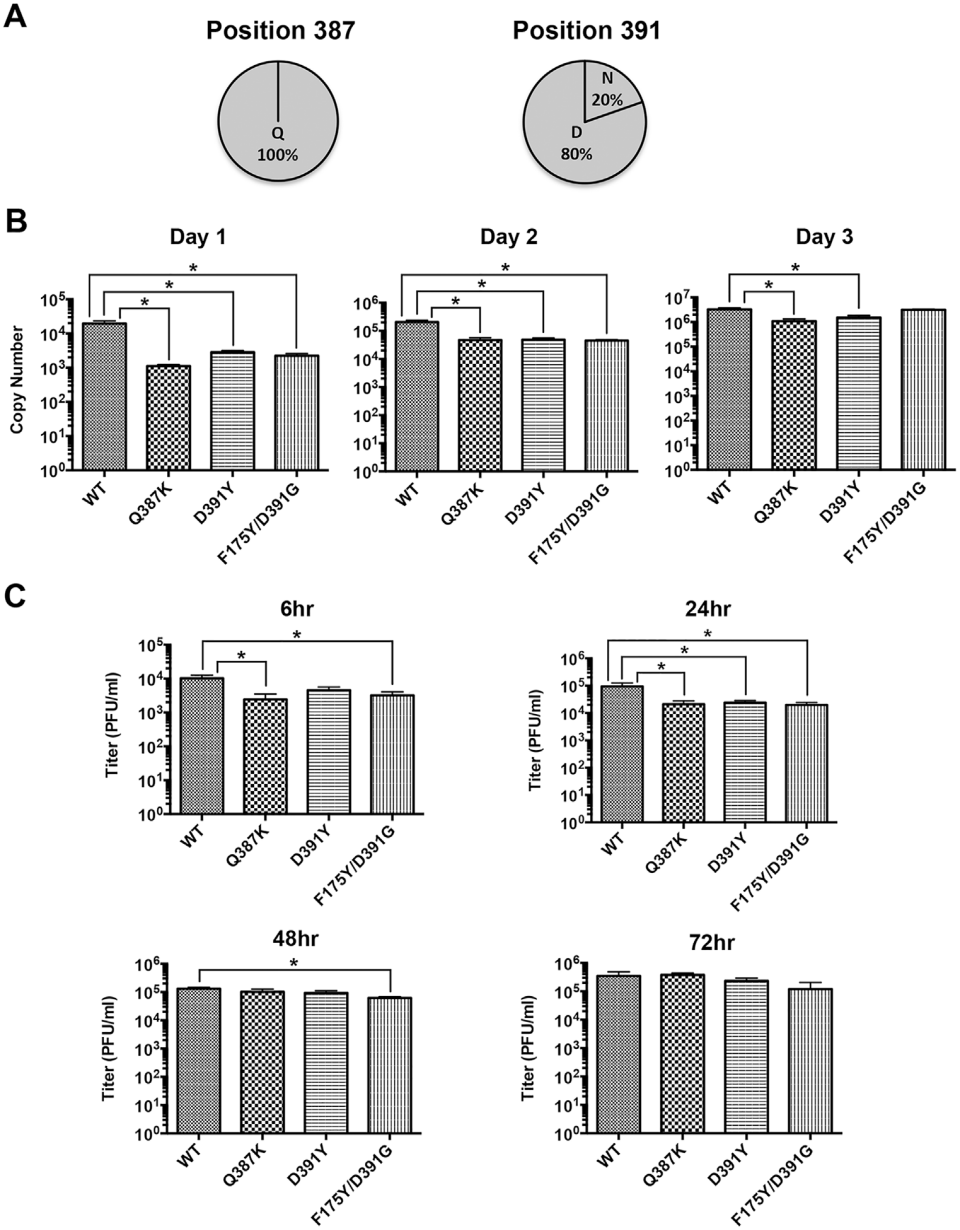
Reduced viral fitness of the A/Perth/16/2009 resistant viruses. (A) A multiple sequence alignment of 11,981 HA amino acid sequences from human and zoonotic isolates of the H1–16 subtypes was used to assess the genetic diversity at positions corresponding to H3 HA 387 and 391. The results are shown as pie charts. (B) In vitro fitness. MDCK cells were infected with the WT or 39.29-resistant A/Perth/16/2009 viruses at MOI 0.01. Released viral genome in the supernatant was quantitated daily by qPCR of the viral M1 Matrix gene. Genome copy numbers per 50 μl of supernatant are shown as histograms. The assay was done in triplicate with data presented as Mean +/- SEM. Statistics were calculated between WT and each of the mutant viruses using a multiple t test with the Prism 6.0 software (* P ≤ 0.05, indicating significant difference). (C) In vivo fitness. DBA/2J mice were infected with same dose of WT or 39.29-resistant A/Perth/16/2009 viruses. At 6 hr, 24 hr, 48 hr and 72 hr post-infection, lung homogenates were prepared and viral titers in the homogenates were determined on MDCK cells. Each group at each time point contained 5 mice. Lung titers were presented as Mean +/- SEM. Statistics were calculated between WT and each of the mutant viruses using a multiple t test with the Prism 6.0 software (* P ≤ 0.05, indicating significant difference).

Since resistance is a paramount concern for therapeutic antibody development, we further characterized the three resistant viruses. First, we performed large multiple sequence alignments [33] to examine whether the Q387K, D391Y and D391G mutations are pre-existing within field isolates of the influenza A virus. We analyzed ~12000 HA sequences from 16 HA subtypes (H1–H16) of influenza A (Fig 8A). Strikingly, Gln387 is 100% conserved among all isolates. At position 391, 80% of the isolates contain an Aspartate (Asp) and the remaining 20% have an Asparagine (Asn).

The highly conserved nature of these two positions suggests that the fitness of the mutant viruses we obtained by antibody selection in vitro might be reduced compared to the WT virus. To test this possibility, we infected MDCK cells with WT or mutant viruses at a low multiplicity of infection (MOI) of 0.01 and measured the amount of released viral genome in the supernatant by qPCR. At day 1 and day 2 post-infection, the three mutant viruses yielded 4–17 fold fewer copies of progeny viral genome compared to the WT virus. At day 3 when all MDCK cells were dead by cell lysis and maximal virus release was reached, the difference between the WT and mutant viruses decreased (Fig 8B). We next tested the in vivo fitness of these viruses in DBA/2J mice. Although the A/Perth/16/2009 virus did not cause lethal infections in mice at any dose tested, lung viral titer increased gradually from 6 hr to 72 hr post-infection suggesting that A/Perth/16/2009 could replicate in the lungs of DBA/2J mice (S5A Fig). The overall lung viral titers were lower than those obtained with mouse-lethal viral strains, consistent with minimal body weight loss at any time point (S5B Fig). Interestingly, all three mutant viruses had lower lung titers than the WT virus at 6 hr and 24 hr, indicating reduced viral fitness in vivo. The difference in lung titers between the WT and mutant viruses decreased at 48 hr and 72 hr, mimicking the in vitro replication results (Fig 8B and 8C). The data indicate that mutant viruses propagate more slowly than WT, but they were still capable of achieving productive replication, approaching WT level after prolonged incubation with susceptible cells. In support of this, none of the mutant viruses reverted to WT or obtained secondary mutations in HA after 10 rounds of sequential passage on MDCK cells in the absence or presence of 39.29.

**Fig 9:**
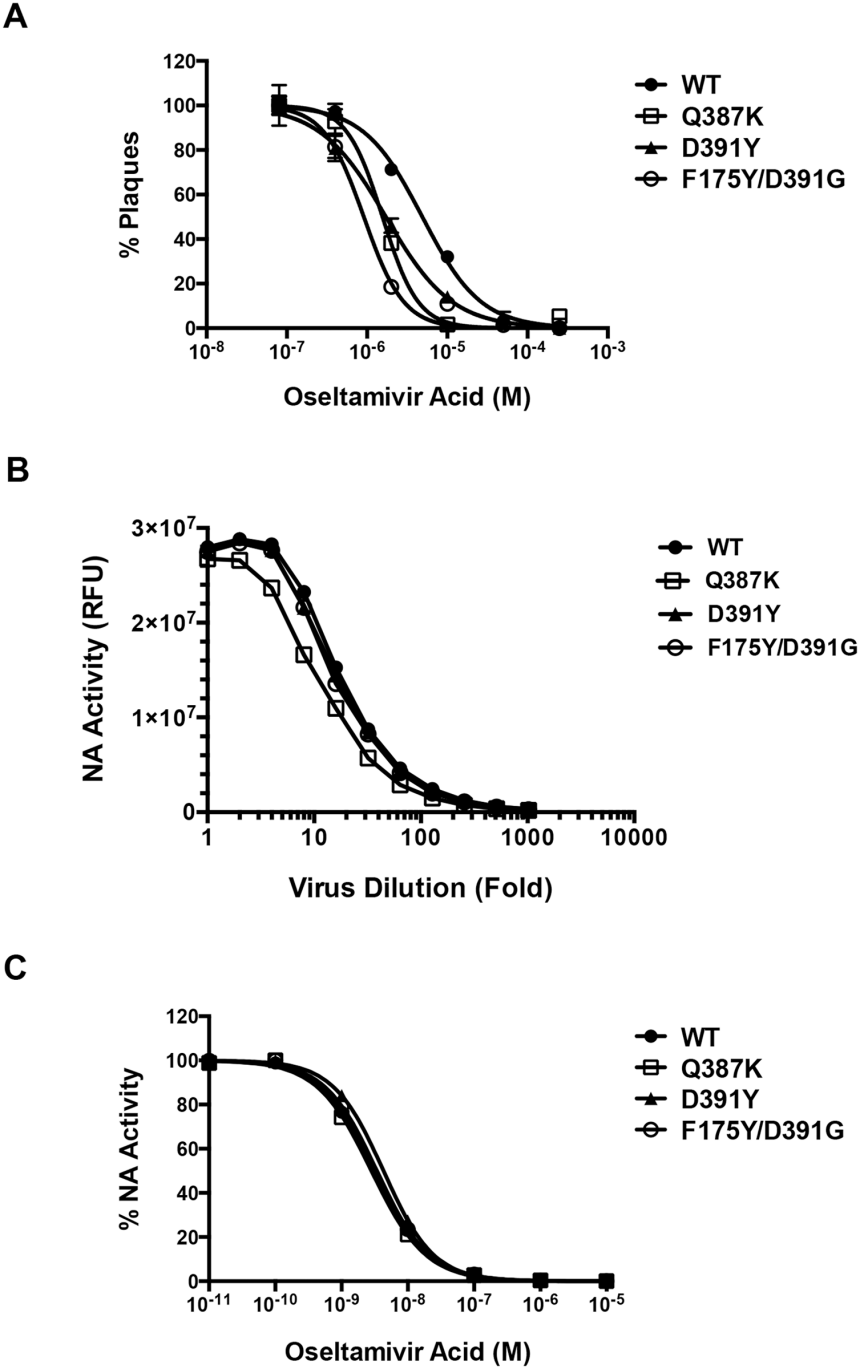
Effect of oseltamivir acid on A/Perth/16/2009 WT and mutant viruses. (A) Plaque reduction assay. MDCK cells in 6-well plates were infected with the WT or 39.29-resistant A/Perth/16/2009 viruses at 100 pfu/well for 1 hour. After removal of the virus inoculum, cells were overlaid with varying concentrations of oseltamivir acid in agarose. The numbers of plaques were counted for each virus and normalized to the number at the lowest oseltamivir acid concentration. The assay was done in triplicate with data presented as Mean +/- SEM. (B) Neuraminidase (NA) activity assay. Serial dilutions of the WT and 39.29-resistant viruses were incubated with a fluorescent NA substrate. NA activities as a function of the fluorescence intensities in relative fluorescence unit (RFU) were plotted on the y-axis versus the log10 virus dilutions on the x-axis. The assay was done in duplicate with data presented as Mean +/- SEM. (C) Virus dilutions with equal NA activities were incubated with varying concentrations of oseltamivir acid, followed by NA activity determination with the fluorescent NA substrate. The NA activities of each virus were normalized to the value at the lowest oseltamivir acid concentration. The assay was done in duplicate with data presented as Mean +/- SEM.

We further attempted to test which step(s) of the viral life cycle are impaired by the mutations. Because one of the current standards of treatment for influenza infection is oseltamivir phosphate (Tamiflu), a small molecule inhibitor of influenza virus release [8], we tested the sensitivity of the WT and mutant viruses to oseltamivir acid (the active form of oseltamivir phosphate) in a plaque reduction assay. Plaque formation of both WT and mutant viruses was effectively inhibited by oseltamivir acid in a dose dependent manner (Fig 9A), suggesting that resistance to 39.29 does not confer cross-resistance to Tamiflu. Interestingly, all three mutant viruses were more sensitive to oseltamivir acid than WT with 3–5 fold lower IC_50_ values (Table 2). Since the viral target of oseltamivir acid is the neuraminidase (NA) protein, which catalyzes the cleavage of the viral receptor sialic acid and thus facilitates the release of progeny viruses from infected cells, we examined the NA function of the WT and mutant viruses. Results from an NA activity assay demonstrated that the NA proteins of the WT and mutant viruses had similar enzymatic activities (Fig 9B), which were similarly inhibited by oseltamivir acid (Fig 9C). These results suggest that the viral production defect of the mutant viruses might not be related to their NA function and that the increased sensitivity of these mutant viruses to oseltamivir acid is likely due to their decreased overall fitness.

**Table 2:** Plaque reduction assay of viruses treated with oseltamivir acid.

Virus	IC_50_ (μM)	95% CI (μM)
**WT**	**4.9**	**4.0–5.9**
**Q387K**	**1.6**	**1.3–1.8**
**D391Y**	**1.7**	**1.4–2.2**
**F175Y/D391G**	**0.9**	**0.7–1.2**

The abbreviations used in the table are as follows: IC_50_, half-maximal inhibitory concentration; CI, confidence interval.

We next performed whole genome sequencing of the WT and resistant viruses in order to identify mutations in viral proteins other than HA that might contribute to the reduced fitness of the resistant viruses. Satisfactory depth of sequencing coverage was achieved for all eight genomic segments of the viruses (S6 Fig). Results of dominant mutations compared to GenBank reference sequences are summarized in Table 3. All mutations in HA were confirmed with no additional changes. For other viral proteins, two unique single amino acid polymorphisms were identified for the WT virus, one in NA (T148K) and the other in NP (G384R). The T148K substitution in NA has been found in recent H3N2 influenza A isolates [34,35] and influenza B isolates [36]. It contributes to reduced inhibition by neuraminidase inhibitors [34–36], likely by affecting the conformation of the NA catalytic site [34]. It is not clear whether this substitution contributes to the reduced sensitivity of the WT virus to oseltamivir acid (Table 2) as WT and mutant viruses have similar NA activities (Fig 9B), which were similarly inhibited by oseltamivir acid (Fig 9C). The R384 residue in WT NP is the anchor residue of a cytotoxic T-lymphocyte (CTL) epitope [37]. The R384G substitution has been found in influenza A viruses isolated from 1993 onwards and allows escape from CTL recognition [37,38]. However, it is detrimental to viral fitness [39]. Therefore, the R384G substitution in all three 39.29-resistant A/Perth/16/2009 viruses possibly contributes to their reduced fitness. There are other none-HA mutations that are unique to one of the three resistant viruses (Table 3). Among these, only the N228I mutation in PA has been reported, and it has no effect on viral fitness [40]. Whether the other mutations contribute to the reduced fitness of the resistant viruses is not known.

**Table 3:** Summary of whole genome sequencing results of A/Perth/16/2009 WT and resistant viruses.

Gene	AA Pos	Ref AA	WT	Q387K	D391Y	F175Y/D391G
**HA**	175	F	-	-	-	Y:99.3%
**HA**	387	Q	-	K:99.3%	-	-
**HA**	391	D	-	-	Y:99.2%	G:99.1%
**NA**	148	T	K:99.3%	-	-	-
**NP**	85	A	-	T:99.3%	-	-
**NP**	384	G	R:99.3%, G:0.6%	-	-	-
**NS1**	112	E	-	K:99.1%	-	-
**NS1**	146	L	-	-	F:78.3%, L:21.1%	-
**PA**	45	C	-	-	-	*:89.3%, C:10.2%
**PA**	46	F	-	-	-	V:87.8%, F:12.0%
**PA**	211	M	-	-	-	I:99.2%
**PA**	228	N	-	-	-	I:98.8%, V:0.5%
**PA**	575	M	-	-	-	L:60.3%, M:39.7%
**PA-X**	45	C	-	-	-	*89.3%, C:10.2%
**PA-X**	46	F	-	-	-	V:87.8%, F:12.0%
**PA-X**	211	C	-	-	-	F99.0%
**PA-X**	228	I	-	-	-	F:99.3%
**PB2**	88	R	-	-	K:70.8%, R:28.6%	-
**PB2**	686	V	-	-	-	M:59.7%, V:39.8%
**Unknown**	57	M	-	-	-	I:99.2%
**Unknown**	74	N	-	-	-	I:98.8%, V:0.5%
**Unknown**	421	M	-	-	-	L:60.3%, M:39.7%

Amino acid substitutions detected at the dominant level (> 50%) in the 3 resistant viruses with respect to the dominant genotype found in the WT virus. Depth of sequencing coverage at positions listed ranges between 1600x to 160000x. Dash indicates no variants detected at a position with respect to the Ref AA. Star indicates nonsense mutation. The abbreviations used in the table are as follows: AA Pos: amino acid position; Ref AA: reference amino acid from GenBank.

Taken together, we discovered three resistant viruses that escaped a broadly neutralizing stalk-binding antibody by two different mechanisms: abolishing antibody binding and enhancing membrane fusion. However, the mutant viruses displayed impaired replication and were highly sensitive to oseltamivir acid suggesting that viral escape in the clinic could be controlled by co-administering neutralizing HA-stalk antibodies with a neuraminidase inhibitor. Of course, careful resistance surveillance during clinical development of HA-stalk antibodies is of highest importance.

## Discussion

Broadly neutralizing stalk-binding mAbs (bnAbs) hold high promise in influenza therapy and the potential of developing a universal flu vaccine. These bnAbs fall into three classes: a) Group 1-specific bnAbs, b) Group 2-specific bnAbs, and c) pan-IAV bnAbs. The epitopes of these bnAbs have been defined. However, extensive studies on the frequency of escape variants and the underlying mechanism of resistance are still needed to address potential liabilities of these antibodies as therapeutics and these epitopes for vaccine development. To-date, antibody-resistant variants have been isolated and characterized only for anti-IAV group 1 and group 2 bnAbs [20–23,27]. Here we report for the first time the isolation of three escape variants to a pan-IAV bnAb. The three variants represent two different escape mechanisms: (a) abolishing antibody binding, which is a common virus escape mechanism, and (b) enhanced membrane fusion ability of HA in conjunction with reduced antibody binding at low pH. To our knowledge, our study is the first report of IAV variants that can escape stalk-binding bnAb by increasing their fusogenic ability to counteract a fusion-inhibiting antibody.

Our binding studies revealed differential binding properties of 39.29 to the three mutants at pH 7 and 4.8. The Q387K mutant completely disrupts binding at neutral or low pH; consequently, this virus is likely endocytosed without bound antibody and achieves productive infection despite a slightly decreased fusion ability compared to the WT (Fig 3). By contrast, the D391Y and F175Y/D391G mutants bind 39.29 as well as the WT at neutral pH, so they are likely internalized into early endosomes with bound antibody [31]. Their binding to 39.29 decreases at pH 4.8 and the affinities are 2–3 fold lower than that of the WT (Table 1). Consequently, when these viruses are within the low pH (5.5–4.6) environment of late endosomes and lysosomes [41], reduced antibody binding may contribute to their escape. The fact that we only isolated resistant variants with mutations in the 39.29 epitope but not the previously identified fusion-enhancing variants with mutations outside helix A and throughout the entire HA ecto-domain [41] supports a role of reduced antibody binding in their escape. For example, the G234E mutant HA demonstrated significantly increased fusion ability but was still blocked by 39.29 because it did not lose antibody binding at low pH (Fig 7). On the other hand, reduction of binding at low pH alone is probably not enough for antibody escape because the same mutations when introduced into an H1 HA (Q386K and D390G) were able to reduce 39.29 binding but could not overcome the fusion block by 39.29, likely due to the lack of fusion-augmenting effect of these mutations in H1 HA (Fig 6). This is consistent with a stoichiometry study of a bnAb CR8020 and an H3N2 virus that the antibody only needs to cover 38% of the HA trimers on the virion to achieve fusion inhibition [30]. Given the high concentration of 39.29 we used during the late rounds of resistance passage, a mild reduction in antibody binding should not significantly impair the ability of the antibody to block membrane fusion and result in virus escape. As an example, a Thr32Arg mutation on HA2 of recent H3N2 strains reduced binding to the anti-IAV group 2 antibody CR8043 by ~10 fold, but these viruses were still potently neutralized by CR8043 [22]. Interestingly, a variant that harbored a His111Leu mutation in a short helix of HA2, which switches from a coiled coil to a reverse turn during fusion [42,43], and reduced binding to the anti-IAV group 1 antibody CR6261 by 60% was able to escape neutralization [27,42,43]. The His111 has previously been identified as important in triggering membrane fusion [44]. It will be interesting to test whether the His111Leu mutation can enhance membrane fusion in addition to partially reducing antibody binding. Taken together, our results suggest that both enhanced fusion ability and reduced antibody binding at low pH are required for the D391 mutants to escape 39.29. Consequently, viral escape to 39.29 is likely a rare event that was only detected for the A/Perth/16/2009 virus. In support of this, we passaged two other IAVs, A/PR/8/1934 (H1N1) and A/Port Chalmers/1/1973 (H3N2), for 17 rounds in the presence of 39.29 (up to concentrations of 6x IC90 for A/PR/8/1934 and 4x IC90 for A/Port Chalmers/1/1973), and no resistant virus emerged.

The D391Y and F175Y/D391G mutations raise the fusion activation pH of HA (Fig 3). It has been shown that stalk-binding antibody traps virus in late endosomes, eventually leading to virus degradation [31]. Fusion at higher pH can help the incoming virus avoid a long stay in the hazardous environment of late endosomes/lysosomes and thus increase the chance of escape. Otterstrom et al. proposed that in order to achieve membrane fusion, an inter-HA network with 2–3 adjacent HA trimers need to be activated simultaneously during the time window of hemifusion (i.e. 30–40 seconds for an H3N2 virus), and inactivation of any trimer in the network by binding of neutralizing antibody will lead to fusion inhibition [30]. Based on this model, several factors might contribute to the escape of D391Y and F175Y/D391G: (1) the reduced binding affinity of D391Y and F175Y/D391G HAs to 39.29 at low pH might give the HAs an elongated window of antibody-free state during virus trafficking from early to late endosomes, although this alone is probably not sufficient for virus escape; (2) elevated fusion activation pH might give the viruses a larger fusion window in endosomes with higher pH; (3) the faster fusion kinetics might increase the chance of productive semifusion before HA inactivation. Since membrane fusion in the presence of neutralizing antibody is a competition between HA inactivation by antibody binding and the fusion ability of HA, all these factors likely contribute to the eventual escape of D391Y and F175Y/D391G. Consequently, combined treatment with 39.29 and an inhibitor of endosomal acidification such as amantadine or bafilomycin A1 [7] might increase the efficacy of 39.29 against these variants. The Q387K mutation, on the other hand, appears to reduce but not abolish the membrane fusion ability of HA at low pH, and mutant viruses harboring the Q387K mutation escape antibody neutralization simply by abolishing antibody binding.

It has been shown that IAV can escape antibody neutralization via mutations in HA that do not alter antibody binding. Yewdell et al. showed that at sub-saturating levels, a mixture of head mAbs selected escape variants harboring single mutations in HA that increased receptor binding while not decreasing antibody binding [12]. Hensley et al. further demonstrated that receptor binding avidity drives IAV antigenic drift based on the results from an elegant study in which IAV was passaged sequentially in immunized and naïve mice [11]. In these studies, adsorptive mutants with enhanced receptor binding ability were selected in the presence of head antibodies that block receptor binding. Similarly, we selected mutants with enhanced fusion ability in the presence of sub-neutralization levels of a stalk antibody that blocks fusion. Hensley et al. also showed that when the adsorptive mutants were passaged in naïve mice, secondary mutations arose decreasing the receptor binding avidity because high receptor binding avidity would interfere with progeny virus release [11]. When we passaged the 39.29-resistant viruses in cell culture for ten rounds (3–4 days per round) in the absence of the antibody, however, no reversion or secondary mutation emerged. At least two factors could contribute to the lack of reversion or secondary mutation. First, although the 39.29-resistant viruses propagate more slowly than WT, they are still capable of achieving productive replication, approaching WT level after prolonged (e.g. 3 days) incubation with susceptible cells. Second, while high receptor binding avidity can interfere with virus release, enhanced fusion ability is only detrimental to the virus at low pH via premature inactivation of HA. When we passaged the viruses in cell culture at neutral pH, the instability of HA caused by the mutations might not contribute to reduced viral fitness. Because mammalian airway tissue is acidic [41], passage of these mutant viruses in mice can potentially lead to reversion or secondary mutations. This is also important in human therapy as potential fusion-enhancing mutants resulted from antibody treatment might be inactivated when transmitted to the airway of untreated people.

Mutations that elevate the fusion activation pH have been discovered in the HA globular head and the stalk region. In general, these include mutations that disrupt intra- and inter-chain interactions and destabilize the secondary and tertiary structures of regions involved in the dramatic conformational change during the fusion process [41]. Escaping variants selected with an anti-HA head antibody HC63 harbor mutations that elevate fusion activation pH by disturbing the HA1-HA1 subunit interface [45]. Interestingly, the mutants could still bind HC63, suggesting an escape mechanism that includes increased fusion ability in conjunction with altered receptor-binding properties. Another fusion-augmenting variant selected for its ability to grow in amantadine by the same group of authors contains a Gln47Arg mutation in the helix A of HA2 [46]. This residue is in close vicinity of the positively charged amino terminus of the fusion peptide on the adjacent HA monomer, and the substitution of a positively charged Arg would result in an electrostatically repulsive effect and thus destabilize the fusion peptide leading to higher fusion ability. The D391 residue in A/Perth/16/2009 HA is #46 in HA2, right next to the Gln47 in helix A and might also be in close distance with the amino terminus of the fusion peptide on the adjacent monomer. Interaction between the positively charged terminal amine group and the negatively charged D391 would possibly stabilize the fusion peptide, and substitution of D391 with neutrally charged Tyr or Gly might abolish this interaction and destabilize the fusion peptide leading to elevated fusion ability. Furthermore, structural analysis showed that D391 forms an intra-helix hydrogen bond with Q387 even when HA is bound by 39.29 (Fig 1B). This interaction is highly conserved among 50 HA structures we examined. Interestingly, Q387 would likely interact with either Aspartate or an isosteric Asparagine side chain at position 391, which is the only other amino acid substitution found at this position in natural isolates (Fig 7A). The hydrogen bond interaction between Q387 and D391 or N391 would likely impart stability to helix A. Consequently, mutation of D391 could destabilize helix A and raise the fusion activation pH. On the other hand, Q387K introduces a positively charged side chain that may form an electrostatic interaction with the negatively charged D391, and thus further stabilize helix A and lower the fusion activation pH (Fig 3). Interestingly, Henry Dunand et al. reported three escape mutants of the highly pathogenic A/Shanghai/1/2013 (H7N9) virus against three potent broadly neutralizing stalk-binding mAbs [23]. Two of the mutant viruses escape by abolishing antibody binding whereas the third one harbors a mutation (I384T in H7 numbering) that only partially reduces antibody binding. The I384 residue is number 390 in H3 numbering, right next to D391. The I384T mutation can possibly disrupt the hydrogen bond between Q387 and D391 and thus destabilize Helix A and increase the fusion ability of HA.

F175Y is likely an inconsequential random mutation as it is distant from the 39.29 epitope and does not affect antibody binding or the fusion ability of HA. However, our results do not rule out the possibility that F175Y contributes to viral escape indirectly. Similarly, the three escape mutants discovered by Henry Dunand et al. also contain mutations in both the head group and the stalk of HA, and the head mutations do not affect antibody binding [23]. The Q387K, D391Y and D391G mutations do not exist in natural isolates of IAV, indicating reduced fitness of the escape variants in nature. Indeed, all three variants release fewer progeny viral particles in cell culture and cause lower lung titers in mouse compared to WT. Interestingly, the replication of the variants approaches WT level at later time points (e.g., 3 days) post-infection, indicating impaired replication rate. The delayed replication of the resistant variants would allow a longer treatment window in clinic. The escape variants also showed higher sensitivity to oseltamivir acid compared to WT. The underlying basis for the differential sensitivities is currently unknown. Although the WT virus harbors a T148K mutation in the neuraminidase (NA) protein that has been associated with reduced sensitivity to NA inhibitors in other H3N2 viruses, the NA activities of the WT and resistant A/Perth/16/2009 viruses are comparable, and are similarly inhibited by oseltamivir acid. Whole genome sequencing revealed an R384G substitution in the NP protein of all three resistant variants compared to the WT virus. This substitution is detrimental to the viral fitness of the A/HK/2/1968 (H3N2) virus [39], and might contribute to the reduced fitness of the three 39.29-resistant variants. Further studies are needed to determine the precise step during the life cycle of the escape variants that is impaired leading to the defective phenotype. The use of re-assortant viruses made from reverse genetics might help understand the effect of each mutation identified by whole genome sequencing on viral fitness. Our data indicate that co-administering stalk-binding bnAbs with a neuraminidase inhibitor would diminish both mechanisms of stalk antibody escape reported here. In conclusion, our studies demonstrate the need to consider novel escape mechanisms, such as increased membrane fusion ability, when studying newly emerged escape variants to these bnAbs.

## Materials and Methods

### Ethics statement

All animals used in this study were housed and maintained at Genentech in accordance with American Association of Laboratory Animal Care guidelines. All experimental studies were conducted under protocols approved by the Institutional Animal Care and Use Committee of Genentech Lab Animal Research in an Association for Assessment and Accreditation of Laboratory Animal Care International-accredited facility in accordance with the Guide for the Care and Use of Laboratory Animals and applicable laws and regulations.

### Isolation of resistant viruses

Madin-Darby Canine Kidney Epithelial (MDCK) cells (ATCC) were grown in DMEM + 10% fetal bovine serum (FBS) in 24-well tissue culture plates. When confluent, cells were incubated with 39.29 at 1x IC50 (half-maximal inhibitory concentration) or 2x IC50 in Flu Media (DMEM + 0.2% BSA + Pen/Strep/Glu + 2 μg/ml TPCK treated Trypsin). A/California/7/2009 (H1N1) or A/Perth/16/2009 (H3N2) virus (ViraPur) was then added at a multiplicity of infection (MOI) of 0.5 in Flu Media. Virus passaged in the absence of antibody is defined as “WT” virus. Every 3 or 4 days, roughly half of the supernatant was passed onto new cells and the concentration of antibody was increased 1.5-fold or held steady so as to maintain virus propagation. As a control, A/California/7/2009 was also passaged in the presence of a neutralizing antibody against the head group of A/California/7/2009 HA (Creative BioMart Cat# CAB-730RI). Viruses were exposed to increasing concentrations of antibody to a final concentration of 1x IC90 (concentration that inhibits 90% of infection) for A/Perth/16/2009 and 4x IC90 for A/California/7/2009. Under these high antibody concentrations, wells containing non-resistant viruses showed minimal cytopathic effect whereas wells containing resistant viruses caused massive cell killing, which can be easily distinguished by the color of the culture medium. Resistant viruses that emerged were stocked without antibody and analyzed for resistance by neutralization assay. RNAs were isolated from true resistant viruses and subjected to reverse transcription, PCR amplification and sequencing. Two additional IAV strains, A/PR/8/1934 (H1N1) and A/Port Chalmers/1/1973 (H3N2), were passaged in the presence of 39.29 (up to concentrations of 6x IC90 for A/PR/8/1934 and 4x IC90 for A/Port Chalmers/1/1973) for 17 rounds, but no resistant virus emerged.

To test the fitness of the A/Perth/16/2009 resistant viruses, they were passaged in the absence or presence of 1x IC90 of 39.29 for 10 rounds. RNAs were extracted from the passaged viruses after each round and subjected to reverse transcription, PCR amplification and sequencing.

### Neutralization assay

MDCK cells were grown as a 50% confluent monolayer in 96-well black imaging plates with clear bottom (Costar). Wild type (WT) or mutant A/Perth/16/2009 virus was diluted in Flu Media to an MOI of 1 and incubated for 1 hour at 37°C with varying concentrations of 39.29. The antibody/virus cocktail was allowed to infect the MDCK cells for 16 hours at 37°C in a 5% CO_2_ incubator prior to fixation with cold 100% ethanol. The fixed cells were then stained with Hoechst 33342 (Invitrogen Cat# H3570) to visualize cell nuclei and determine total cell number. The cells were also stained with a monoclonal antibody specific for IAV nucleoprotein (NP) (Millipore Cat# MAB8258) to determine the number of infected cells. Cells were imaged using an Image Express Micro apparatus (Molecular Devices) and data images were analyzed using the software MetaXpress 3.1. The percentages of infected cells (normalized to the lowest antibody concentration) were determined and plotted on the Y-axis versus the Log10 antibody concentrations on the X-axis. The data were fit with a nonlinear regression dose response curve using the GraphPad Prism v.6.0 software.

### Flow cytometry

Human embryonic kidney epithelial (293T) cells (ATCC) were grown in DMEM + 10% FBS in 100mm tissue culture dishes. Cells were transfected with 30 μg of plasmid encoding the WT or mutant HA or mock transfected using the calcium phosphate method. Two days later, cells were collected in a PBS-based enzyme-free cell dissociation buffer (Life Technologies) and blocked with PBS + 5% FBS. Cells were stained with 39.29 or a positive control antibody 36.94 that binds to all IAV HAs tested [25] in PBS (pH 7) + 5% FBS or a citric acid/sodium citrate buffer (pH 4.8) + 5% FBS. Cells were washed and then stained with a DyLight 649-conjugated anti-human secondary antibody (Jackson ImmunoResearch). Stained cells were analyzed on a FACSCalibur (BD Biosciences). Results were plotted with the FlowJo 8.4.5 software.

### Equilibrium binding (Scatchard) assay

293T cells were grown in DMEM + 10% FBS in 100mm tissue culture dishes. Cells were transfected with 30 μg of plasmid encoding the WT or mutant HA using the calcium phosphate method. Two days later, cells were collected in a PBS-based enzyme-free cell dissociation buffer. Binding affinities of 39.29 or 36.94 to surface-expressed HAs were determined by equilibrium binding analysis where ^125^I‑labeled antibodies and varying concentrations of unlabeled antibodies were incubated with the cells at room temperature (RT) for 2 hours. Radioactivity signals of the samples were measured on a Wizard-2 Wallac 2470 Counter (PerkinElmer). The binding affinities were derived from the displacement binding data using the NewLigand software (Genentech).

### Immunogold-negative staining electron microscopy (EM)

WT and mutant A/Perth/16/2009 viruses in suspension were adsorbed for 15 minutes to the surface of transmission electron microscope (TEM) grids. Grids were blocked with a blocking solution (Aurion Electron Microscopy Sciences) for 30 minutes followed by incubation with either a positive control antibody or 39.29 for 1 hour at RT. Virus particles bound by the positive control antibody were detected by a 6 nm gold-conjugated secondary antibody (Jackson ImmunoResearch); virus particles bound by 39.29 were detected by a biotinylated secondary antibody (Jackson ImmunoResearch) and a streptavidin 10 nm gold-conjugate (Invitrogen). Finally, grids were washed, treated with 4% paraformaldehyde to inactivate all virus particles and counter stained with Nano-W (Nanoprobes). Samples were examined under a JEOL JEM-1400 TEM at 120kV. Digital images were captured with a GATAN Ultrascan 1000 CCD camera at magnifications from 1000X to 50000X.

### Fusion assays

Hela cells (ATCC) were grown in DMEM + 10% FBS to ~ 40% confluent in 6-well tissue culture plates and transfected with 5 μg of plasmid encoding the WT or mutant HA. Two days later when cells were 100% confluent, they were washed with PBS and treated with 5 μg/ml of TPCK treated trypsin (Sigma) in PBS for 5 minutes at 37°C. Trypsin was removed and cells were incubated with culture media containing 25 μg/ml of soybean trypsin inhibitor (CalBiochem) for 1 hour at 37°C. Cells were then incubated with a citric acid/sodium citrate buffer of various pHs (4.8 to 6.0) for 2 minutes at 37°C to induce fusion. Cells were washed with DMEM and grow in culture media overnight to allow full formation of syncytia. Phase images of the cells were acquired under the 10X objective on a Nikon Eclipse TE2000-E microscope with the NIS-Elements AR3.2 software. In some experiments, trypsin treated cells were incubated with 200 μg/ml of 39.29 or a negative control mAb against the gD glycoprotein of cytomegalovirus before the pH drop.

To quantitate the fusion results, Hela cells were cotransfected with an HA-expressing plasmid (or the empty plasmid as negative control) and a pTRE-Luciferase plasmid (Clontech) that carries a luciferase gene under the control of a tetracycline (Tet)-inducible promoter. In parallel, Hela Tet-On 3G cells (Clontech), which stably express the Tet-On transactivator, were transfected with the HA-expressing plasmid (or the empty plasmid as negative control). The cells were mixed at a 1:1 ratio and subjected to the trypsin treatment and pH drop as above. For the time-course experiment, cells were incubated with a buffer of pH 5.7 for 20, 40, 60 or 120 seconds before medium change. After overnight incubation, cells were lysed following the instructions of the One-Glo Luciferase Assay System (Promega) and the luminescence signals in the lysates were measured on an EnVision plate reader (PerkinElmer).

### HA conformational change assay by flow cytometry

293T cells were transfected with plasmid encoding the WT or mutant HA or mock transfected. Two days later, cells were collected in a PBS-based enzyme-free cell dissociation buffer and washed with PBS. A fraction of cells were set aside as Stage 1 samples. Cells were then treated with 3 μg/ml of TPCK treated trypsin in PBS for 2 minutes and neutralized with 25 μg/ml of soybean trypsin inhibitor. Again, a fraction of cells were set aside as Stage 2 samples. The remaining cells were incubated with 39.29 for 1 hour and then treated with a citric acid/sodium citrate buffer of pH 4.8 for 2 minutes at 37°C to induce HA conformational change. Cells were washed and set aside as Stage 3 samples. Samples at all three stages were tested for 39.29 binding with a DyLight 649-conjugated anti-human secondary antibody. Stained cells were analyzed on a FACSCalibur and results were analyzed with the FlowJo 8.4.5 software. The mean fluorescence intensities of the samples at each stage were normalized to the WT and the percentages of binding were plotted as histograms using the GraphPad Prism v.6.0 software. Fractions of Stage 1 and Stage 2 cells were also subjected to Western blot analysis to test trypsin cleavage efficiency.

### Western blot analysis

Cells were lysed in Triton Lysis Buffer (50 mM Tris pH8, 5 mM EDTA pH8, 150 mM NaCl, 1% Triton X100 and protease inhibitors). Lysates were centrifuged at 14000 rpm for 10 minutes to pellet nuclei. Supernatants were subjected to SDS-PAGE on a 4–12% NuPAGE gel (Invitrogen). The gel was transferred onto a Nitrocellulose membrane and Western blot analysis was performed with a mouse monoclonal antibody against the HA1 subunit of H3 HA (Millipore #MAB8254) and an IRDye 680LT anti-mouse secondary antibody (Licor). Images were obtained and quantitated with the Odyssey Imaging System (Licor).

### Viral genome quantification in culture supernatant

MDCK cells were grown in 96-well tissue culture plates and infected with the WT or mutant A/Perth/16/2009 virus at an MOI of 0.01. Culture supernatants (50 μl) were collected at day 1, 2 and 3 post-infection and subjected to RNA isolation with an SV 96 Total RNA Isolation System (Promega). cDNA synthesis was carried out with a High Capacity cDNA Reverse Transcription Kit (Applied Biosystems), followed by quantitative real-time PCR (qPCR) using a TaqMan Universal PCR Master Mix (Applied Biosystems) to detect the A/Perth/16/2009 M1 Matrix gene with the following oligonucleotides (5’– 3’):

Sense: AAGACCAATTCTGTCACCTCTGA

Antisense: CAAAGCGTCTACGCTGCAGTCC

Probe: TTTGTGTTCACGCTCACCGT

### Mouse in vivo fitness studies

DBA/2J mice (Jackson Laboratory) were infected intranasally with 1.5x10^6^ plaque forming units (PFU) of A/Perth/16/2009 WT or mutant viruses diluted in 50 μl Flu Media. At 6 hr, 24 hr, 48 hr and 72 hr post-infection, body weight (BW) was recorded before mice were euthanized. Lung after exsanguination was collected in 2 mL Flu Medium and homogenized by using gentleMACS with the RNA01.01 Program. Lung homogenates were spun at 3000 rpm for 10 minutes and then cleared through 70 μm cell strainer. The supernatants were collected on ice and tittered on MDCK cells immediately. Briefly, lung homogenates were incubated with MDCK cells for 16 hours at 37C prior to cell staining as above (under “Neutralization assay”). The numbers of viral positive foci were determined with the MetaXpress 3.1 software and lung titers were plotted as histograms in PFU/ml. BW data were plotted as percent BW change compared to pre-infection (0 hr) BW.

### Plaque reduction assay

MDCK cells were grown in 6-well tissue culture plates and infected with serial dilutions of the WT or mutant A/Perth/16/2009 virus in Flu Media. One hour after infection, viruses were removed and cells were overlaid with Flu Media + 1% agarose. Infected cells were incubated at 37°C for 3 days and the numbers of plaques were counted under microscope. For each virus, the amount of viral stock that gives ~100 plaques (100 plaque forming units or 100 pfu) was used for plaque reduction assay. Briefly, cells were infected with WT or mutant virus for 1 hour. After removal of the virus inoculum, cells were overlaid with varying concentrations of oseltamivir acid (Toronto Research Chemicals Cat# O700980) in Flu Media + 1% agarose and incubated at 37°C until plaques were visible. The numbers of plaques were counted for each virus at each drug concentration. The percentages of plaques (normalized to the numbers at the lowest drug concentration) were determined and plotted on the Y-axis versus the Log10 concentrations of oseltamivir acid on the X-axis. The data were fit with a nonlinear regression dose response curve using the GraphPad Prism v.6.0 software.

### Neuraminidase activity assay

The neuraminidase (NA) activities of the viruses were measured with an NA-Fluor^™^ Influenza Neuraminidase Assay Kit (Applied Biosystems). Briefly, serial dilutions of the viruses in 96-well black-wall clear-bottom imaging plates were incubated with the fluorescent NA substrate (4-methylumbelliferyl-N-acetylneuraminic acid) at 37°C for 1 hour prior to assay termination by addition of 100 μl of the NA-Fluor stop solution. The fluorescence intensities were measured on an EnVision plate reader (PerkinElmer). The NA activities as a function of the fluorescence intensities in relative fluorescence unit (RFU) were plotted on the y-axis versus the log10 virus dilutions on the x-axis. The data were fit with a nonlinear regression dose-response curve to determine the optimal dilution for each virus.

Virus dilutions with equal NA activities were used in the oseltamivir acid inhibition assay. Each virus preparation in duplicate was incubated with varying concentrations of oseltamivir acid for 30 minutes at 37°C. The fluorescent NA substrate was then added and incubated for 1 hour at 37°C prior to assay termination. The fluorescence intensities were measured as above. The NA activities were normalized for each virus and the percentages of NA activities were plotted on the y-axis versus the log10 oseltamivir acid concentrations on the x-axis.

### Sequence alignment

A multiple sequence alignment of 11,981 HA amino acid sequences comprised of human and zoonotic isolates from the H1, H2, H3, H4, H5, H6, H7, H8, H9, H10, H11, H12, H13, H14, H15, and H16 subtypes was used to assess the genetic diversity of the 39.29 contact residues [33]. The alignment was visualized in JalView [47]. Sequence analysis was performed using a customized software pipeline (written in Perl), and entailed numerical assessment of HA subtypes from the alignment. These data were used to generate a frequency table of amino acids observed at each individual position. The results were used to generate pie charts depicting proportions of individual amino acids at the relevant positions corresponding to H3 HA 387 and 391.

### Whole genome sequencing

RNAs were extracted from the A/Perth/16/2009 WT and resistant viruses and subjected to reverse transcription and PCR amplification using the PathAmp FluA Reagents (Thermo Fisher). A fraction of the PCR products were resolved and visualized on 1% agarose gel to ensure the presence of all 8 influenza genome segments (PB2, PB1, PA, HA, NP, NA, M, NS) at the expected sizes (2.31, 2.27, 2.12, 1.70, 1.54, 1.41, 0.98, 0.84 kb). The remaining PCR products were purified using the DNA Clean & Concentrator (Zymo Research), and contracted to SeqWright (GE Healthcare) for library preparation and whole genome sequencing with Illumina paired-end technology (2x100bp) on the Illumina Hiseq platform (Illumina). The genomic alignment software *GSNAP* was used for all FASTQ sequence alignment [48]. GenBank sequences of A/Perth/16/2009 (Accession No. KJ609203—KJ609210) were used as the reference sequences for alignment (S1 Table). Sequence analysis was carried out by Genentech in-house viral bioinformatics pipeline based on R and Bioconductor packages: GenomicRanges [49], GenomicAlignments [49], VariantTools [50], gmapR [51]. Only base-calls with Q-score ≥ 30 were tallied for variant calling. Amino acid substitutions with respect to the reference sequences were determined at a frequency threshold of 50%.

### Crystal structure

The structure of A/Perth/16/2009 HA bound by 39.29 was determined by us previously [25] and can be accessed using the PDB ID, 4KVN. Fig 1B was prepared with PYMOL [52].

## Supporting Information

S1 Fig Similar binding and fusion properties of D391G and F175Y/D391G HAs. (A) 293T cells expressing the WT, F175Y, D391G or F175Y/D391G A/Perth/16/2009 HA were incubated with a positive control antibody (left panel) or 39.29 (middle and right panels) at pH 7 (left and middle panels) or 4.8 (right panel). Flow cytometry profiles are shown. Mock, mock transfected cells. (B) Hela cells expressing the WT, F175Y, D391G or F175Y/D391G A/Perth/16/2009 HA were treated with trypsin to activate HA0 and then incubated with buffers at different pHs for 2 minutes to induce cell-cell fusion. After overnight culture, representative images were obtained under a phase contrast microscope.
(TIF)

S2 Fig Cells expressing no HA do not fuse and give ignorable luminescence signals. Hela cells expressing the WT A/Perth/16/2009 HA or no HA (transfected with the empty plasmid) plus a tetracycline (Tet)-inducible luciferase protein were mixed with Hela Tet-On 3G cells expressing the WT HA or no HA, respectively. Cells were treated with trypsin to activate HA0 and then incubated with buffers at different pHs for 2 minutes to induce cell-cell fusion. After overnight culture, cells were lysed and incubated with a luminescent substrate of the luciferase. Luminescence signals were measured and are shown as histograms in random luminescence units (RLU). The assay was done in triplicate with data presented as Mean +/- SEM.
(TIF)

S3 Fig Trypsin cleavage converts HA0 into HA1/HA2. 293T cells expressing the WT A/Perth/16/2009 HA or the Q387K, D391Y or F175Y/D391G mutant HA were collected before and after trypsin treatment. Cells were lysed in Triton Lysis Buffer and the lysates were subjected to SDS-PAGE and Western blot analysis with an antibody against the HA1 subunit of H3 HA. % HA0 is the band intensity of HA0 divided by the band intensities of HA0 + HA1. As a control, a purified HA protein was digested by trypsin and analyzed under the same conditions.
(TIF)

S4 Fig G234E mutation enhances the fusion ability of A/Perth/16/2009 HA. Hela cells expressing the WT or G234E A/Perth/16/2009 HA were treated with trypsin to activate HA0 and then incubated with buffers at different pHs for 2 minutes to induce cell-cell fusion. After overnight culture, representative images were obtained under a phase contrast microscope.
(TIF)

S5 Fig Lung titers and body weight (BW) changes of mice infected with WT or mutant A/Perth/16/2009 viruses at various time points. (A) DBA/2J mice were infected with WT A/Perth/16/2009 intranasally. At 6 hr, 24 hr, 48 hr and 72 hr post-infection, lung homogenates were prepared and viral titers in the homogenates were determined on MDCK cells. Each time point contained 5 mice. Lung titers were presented as Mean +/- SEM. (B) DBA/2J mice were infected with same dose of WT or 39.29-resistant A/Perth/16/2009 viruses. At 0 hr (pre-infection), 6 hr, 24 hr, 48 hr and 72 hr post-infection, BW was recorded and plotted as percent BW change compared to pre-infection BW. Each group at each time point contained 5 mice. Data were presented as Mean +/- SEM.
(TIF)

S6 Fig Depth of coverage for each segment in the genome of A/Perth/16/209 WT and resistant viruses. (TIF)

S7 Fig Similar membrane fusion properties of WT and mutant HAs between pH 4.8 and 5.5. (A) Hela cells expressing the WT or mutant A/Perth/16/2009 HAs were treated with trypsin to activate HA0 and then incubated with buffers at different pHs for 2 minutes to induce cell-cell fusion. After overnight culture, representative images were obtained under a phase contrast microscope.
(TIF)

S1 Table Genomic coordinates for each gene in the A/Perth/16/2009 reference sequences (GenBank Accession No. KJ609203—KJ609210). (DOCX)

## References

[1] Lozano R, Naghavi M, Foreman K, Lim S, Shibuya K, et al (2012) Global and regional mortality from 235 causes of death for 20 age groups in 1990 and 2010: a systematic analysis for the Global Burden of Disease Study 2010. Lancet 380: 2095–2128. 10.1016/S0140-6736(12)61728-0 23245604 PMC10790329

[2] Thompson WW, Shay DK, Weintraub E, Brammer L, Cox N, et al (2003) Mortality associated with influenza and respiratory syncytial virus in the United States. JAMA 289: 179–186. 12517228 10.1001/jama.289.2.179

[3] Thompson WW, Shay DK, Weintraub E, Brammer L, Bridges CB, et al (2004) Influenza-associated hospitalizations in the United States. JAMA 292: 1333–1340. 15367555 10.1001/jama.292.11.1333

[4] Murray CJ, Lopez AD, Chin B, Feehan D, Hill KH (2006) Estimation of potential global pandemic influenza mortality on the basis of vital registry data from the 1918–20 pandemic: a quantitative analysis. Lancet 368: 2211–2218. 17189032 10.1016/S0140-6736(06)69895-4

[5] Pebody R, Warburton F, Ellis J, Andrews N, Thompson C, et al (2015) Low effectiveness of seasonal influenza vaccine in preventing laboratory-confirmed influenza in primary care in the United Kingdom: 2014/15 mid-season results. Euro Surveill 20. 25677050

[6] Spanakis N, Pitiriga V, Gennimata V, Tsakris A (2014) A review of neuraminidase inhibitor susceptibility in influenza strains. Expert Rev Anti Infect Ther 12: 1325–1336. 10.1586/14787210.2014.966083 25301229

[7] Edinger TO, Pohl MO, Stertz S (2014) Entry of influenza A virus: host factors and antiviral targets. J Gen Virol 95: 263–277. 10.1099/vir.0.059477-0 24225499

[8] Quinones-Parra S, Loh L, Brown LE, Kedzierska K, Valkenburg SA (2014) Universal immunity to influenza must outwit immune evasion. Front Microbiol 5: 285 10.3389/fmicb.2014.00285 24971078 PMC4054793

[9] Lee PS, Wilson IA (2015) Structural characterization of viral epitopes recognized by broadly cross-reactive antibodies. Curr Top Microbiol Immunol 386: 323–341. 10.1007/82_2014_413 25037260 PMC4358778

[10] Xu R, Ekiert DC, Krause JC, Hai R, Crowe JE Jr., et al (2010) Structural basis of preexisting immunity to the 2009 H1N1 pandemic influenza virus. Science 328: 357–360. 10.1126/science.1186430 20339031 PMC2897825

[11] Hensley SE, Das SR, Bailey AL, Schmidt LM, Hickman HD, et al (2009) Hemagglutinin receptor binding avidity drives influenza A virus antigenic drift. Science 326: 734–736. 10.1126/science.1178258 19900932 PMC2784927

[12] Yewdell JW, Caton AJ, Gerhard W (1986) Selection of influenza A virus adsorptive mutants by growth in the presence of a mixture of monoclonal antihemagglutinin antibodies. J Virol 57: 623–628. 2418215 10.1128/jvi.57.2.623-628.1986 PMC252777

[13] Corti D, Lanzavecchia A (2013) Broadly neutralizing antiviral antibodies. Annu Rev Immunol 31: 705–742. 10.1146/annurev-immunol-032712-095916 23330954

[14] Corti D, Suguitan AL Jr., Pinna D, Silacci C, Fernandez-Rodriguez BM, et al (2010) Heterosubtypic neutralizing antibodies are produced by individuals immunized with a seasonal influenza vaccine. J Clin Invest 120: 1663–1673. 10.1172/JCI41902 20389023 PMC2860935

[15] Ekiert DC, Kashyap AK, Steel J, Rubrum A, Bhabha G, et al (2012) Cross-neutralization of influenza A viruses mediated by a single antibody loop. Nature 489: 526–532. 10.1038/nature11414 22982990 PMC3538848

[16] Whittle JR, Zhang R, Khurana S, King LR, Manischewitz J, et al (2011) Broadly neutralizing human antibody that recognizes the receptor-binding pocket of influenza virus hemagglutinin. Proc Natl Acad Sci U S A 108: 14216–14221. 10.1073/pnas.1111497108 21825125 PMC3161572

[17] Yoshida R, Igarashi M, Ozaki H, Kishida N, Tomabechi D, et al (2009) Cross-protective potential of a novel monoclonal antibody directed against antigenic site B of the hemagglutinin of influenza A viruses. PLoS Pathog 5: e1000350 10.1371/journal.ppat.1000350 19300497 PMC2652660

[18] Ekiert DC, Bhabha G, Elsliger MA, Friesen RH, Jongeneelen M, et al (2009) Antibody recognition of a highly conserved influenza virus epitope. Science 324: 246–251. 10.1126/science.1171491 19251591 PMC2758658

[19] Sui J, Hwang WC, Perez S, Wei G, Aird D, et al (2009) Structural and functional bases for broad-spectrum neutralization of avian and human influenza A viruses. Nat Struct Mol Biol 16: 265–273. 10.1038/nsmb.1566 19234466 PMC2692245

[20] Okuno Y, Isegawa Y, Sasao F, Ueda S (1993) A common neutralizing epitope conserved between the hemagglutinins of influenza A virus H1 and H2 strains. J Virol 67: 2552–2558. 7682624 10.1128/jvi.67.5.2552-2558.1993 PMC237575

[21] Ekiert DC, Friesen RH, Bhabha G, Kwaks T, Jongeneelen M, et al (2011) A highly conserved neutralizing epitope on group 2 influenza A viruses. Science 333: 843–850. 10.1126/science.1204839 21737702 PMC3210727

[22] Friesen RH, Lee PS, Stoop EJ, Hoffman RM, Ekiert DC, et al (2014) A common solution to group 2 influenza virus neutralization. Proc Natl Acad Sci U S A 111: 445–450. 10.1073/pnas.1319058110 24335589 PMC3890827

[23] Henry Dunand CJ, Leon PE, Kaur K, Tan GS, Zheng NY, et al (2015) Preexisting human antibodies neutralize recently emerged H7N9 influenza strains. J Clin Invest 125: 1255–1268. 10.1172/JCI74374 25689254 PMC4362269

[24] Corti D, Voss J, Gamblin SJ, Codoni G, Macagno A, et al (2011) A neutralizing antibody selected from plasma cells that binds to group 1 and group 2 influenza A hemagglutinins. Science 333: 850–856. 10.1126/science.1205669 21798894

[25] Nakamura G, Chai N, Park S, Chiang N, Lin Z, et al (2013) An in vivo human-plasmablast enrichment technique allows rapid identification of therapeutic influenza A antibodies. Cell Host Microbe 14: 93–103. 10.1016/j.chom.2013.06.004 23870317

[26] Dreyfus C, Laursen NS, Kwaks T, Zuijdgeest D, Khayat R, et al (2012) Highly conserved protective epitopes on influenza B viruses. Science 337: 1343–1348. 10.1126/science.1222908 22878502 PMC3538841

[27] Throsby M, van den Brink E, Jongeneelen M, Poon LL, Alard P, et al (2008) Heterosubtypic neutralizing monoclonal antibodies cross-protective against H5N1 and H1N1 recovered from human IgM+ memory B cells. PLoS One 3: e3942 10.1371/journal.pone.0003942 19079604 PMC2596486

[28] Ohkuma S, Poole B (1978) Fluorescence probe measurement of the intralysosomal pH in living cells and the perturbation of pH by various agents. Proc Natl Acad Sci U S A 75: 3327–3331. 28524 10.1073/pnas.75.7.3327 PMC392768

[29] Su B, Wurtzer S, Rameix-Welti MA, Dwyer D, van der Werf S, et al (2009) Enhancement of the influenza A hemagglutinin (HA)-mediated cell-cell fusion and virus entry by the viral neuraminidase (NA). PLoS One 4: e8495 10.1371/journal.pone.0008495 20041119 PMC2795206

[30] Otterstrom JJ, Brandenburg B, Koldijk MH, Juraszek J, Tang C, et al (2014) Relating influenza virus membrane fusion kinetics to stoichiometry of neutralizing antibodies at the single-particle level. Proc Natl Acad Sci U S A 111: E5143–5148. 10.1073/pnas.1411755111 25404330 PMC4260548

[31] Brandenburg B, Koudstaal W, Goudsmit J, Klaren V, Tang C, et al (2013) Mechanisms of hemagglutinin targeted influenza virus neutralization. PLoS One 8: e80034 10.1371/journal.pone.0080034 24348996 PMC3862845

[32] Steinhauer DA, Martin J, Lin YP, Wharton SA, Oldstone MB, et al (1996) Studies using double mutants of the conformational transitions in influenza hemagglutinin required for its membrane fusion activity. Proc Natl Acad Sci U S A 93: 12873–12878. 8917512 10.1073/pnas.93.23.12873 PMC24013

[33] Roca AI, Abajian A.C., and Vigerust D.J. (2013) ProfileGrids solve the large alignment visualization problem: influenza hemagglutinin example. F1000Research 2: 1–5.24358860

[34] Tamura D, Nguyen HT, Sleeman K, Levine M, Mishin VP, et al (2013) Cell culture-selected substitutions in influenza A(H3N2) neuraminidase affect drug susceptibility assessment. Antimicrob Agents Chemother 57: 6141–6146. 10.1128/AAC.01364-13 24080660 PMC3837856

[35] Takashita E, Meijer A, Lackenby A, Gubareva L, Rebelo-de-Andrade H, et al (2015) Global update on the susceptibility of human influenza viruses to neuraminidase inhibitors, 2013–2014. Antiviral Res 117: 27–38. 10.1016/j.antiviral.2015.02.003 25721488 PMC9036627

[36] Jonges M, van der Lubben IM, Dijkstra F, Verhoef L, Koopmans M, et al (2009) Dynamics of antiviral-resistant influenza viruses in the Netherlands, 2005–2008. Antiviral Res 83: 290–297. 10.1016/j.antiviral.2009.07.003 19591877

[37] Voeten JT, Bestebroer TM, Nieuwkoop NJ, Fouchier RA, Osterhaus AD, et al (2000) Antigenic drift in the influenza A virus (H3N2) nucleoprotein and escape from recognition by cytotoxic T lymphocytes. J Virol 74: 6800–6807. 10888619 10.1128/jvi.74.15.6800-6807.2000 PMC112197

[38] Berkhoff EG, Boon AC, Nieuwkoop NJ, Fouchier RA, Sintnicolaas K, et al (2004) A mutation in the HLA-B*2705-restricted NP383-391 epitope affects the human influenza A virus-specific cytotoxic T-lymphocyte response in vitro. J Virol 78: 5216–5222. 15113903 10.1128/JVI.78.10.5216-5222.2004 PMC400375

[39] Rimmelzwaan GF, Berkhoff EG, Nieuwkoop NJ, Fouchier RA, Osterhaus AD (2004) Functional compensation of a detrimental amino acid substitution in a cytotoxic-T-lymphocyte epitope of influenza a viruses by comutations. J Virol 78: 8946–8949. 15280506 10.1128/JVI.78.16.8946-8949.2004 PMC479054

[40] Sun HL, Jiao PR, Cheng YQ, Yuan RY, Cui PF, et al (2011) The Pathogenicity Variation of Two Quail-Origin H5N1 HPAV to BALB/c Mice after Six Passages in Quail. Journal of Animal and Veterinary Advances 10: 1974–1980.

[41] Russell CJ (2014) Acid-induced membrane fusion by the hemagglutinin protein and its role in influenza virus biology. Curr Top Microbiol Immunol 385: 93–116. 10.1007/82_2014_393 25007844 PMC7122338

[42] Wilson IA, Skehel JJ, Wiley DC (1981) Structure of the haemagglutinin membrane glycoprotein of influenza virus at 3 A resolution. Nature 289: 366–373. 7464906 10.1038/289366a0

[43] Bullough PA, Hughson FM, Skehel JJ, Wiley DC (1994) Structure of influenza haemagglutinin at the pH of membrane fusion. Nature 371: 37–43. 8072525 10.1038/371037a0

[44] Thoennes S, Li ZN, Lee BJ, Langley WA, Skehel JJ, et al (2008) Analysis of residues near the fusion peptide in the influenza hemagglutinin structure for roles in triggering membrane fusion. Virology 370: 403–414. 17936324 10.1016/j.virol.2007.08.035 PMC2212604

[45] Daniels PS, Jeffries S, Yates P, Schild GC, Rogers GN, et al (1987) The receptor-binding and membrane-fusion properties of influenza virus variants selected using anti-haemagglutinin monoclonal antibodies. EMBO J 6: 1459–1465. 3608984 10.1002/j.1460-2075.1987.tb02387.x PMC553952

[46] Daniels RS, Downie JC, Hay AJ, Knossow M, Skehel JJ, et al (1985) Fusion mutants of the influenza virus hemagglutinin glycoprotein. Cell 40: 431–439. 3967299 10.1016/0092-8674(85)90157-6

[47] Waterhouse AM, Procter JB, Martin DM, Clamp M, Barton GJ (2009) Jalview Version 2—a multiple sequence alignment editor and analysis workbench. Bioinformatics 25: 1189–1191. 10.1093/bioinformatics/btp033 19151095 PMC2672624

[48] Wu TD, Nacu S (2010) Fast and SNP-tolerant detection of complex variants and splicing in short reads. Bioinformatics 26: 873–881. 10.1093/bioinformatics/btq057 20147302 PMC2844994

[49] Lawrence M, Huber W, Pages H, Aboyoun P, Carlson M, et al (2013) Software for computing and annotating genomic ranges. PLoS Comput Biol 9: e1003118 10.1371/journal.pcbi.1003118 23950696 PMC3738458

[50] Lawrence M DJ, Gentleman R (2016) VariantTools: Tools for working with genetic variants. R package version 1.13.3.

[51] Barr C WT, Lawrence M (2016) gmapR: an R interface to the GMAP/GSNAP/GSTRUCT suite. R package version 1.12.0.

[52] The PyMOL Molecular Graphics System V, Schrödinger, LLC.

