# Supplementary figures and images for: Two Escape Mechanisms of Influenza A Virus to a Broadly Neutralizing Stalk-Binding Antibody
Source: PLoS Pathog. 2016 Jun 28;12(6):e1005702. doi: 10.1371/journal.ppat.1005702 (PMC4924800; doi:10.1371/journal.ppat.1005702)

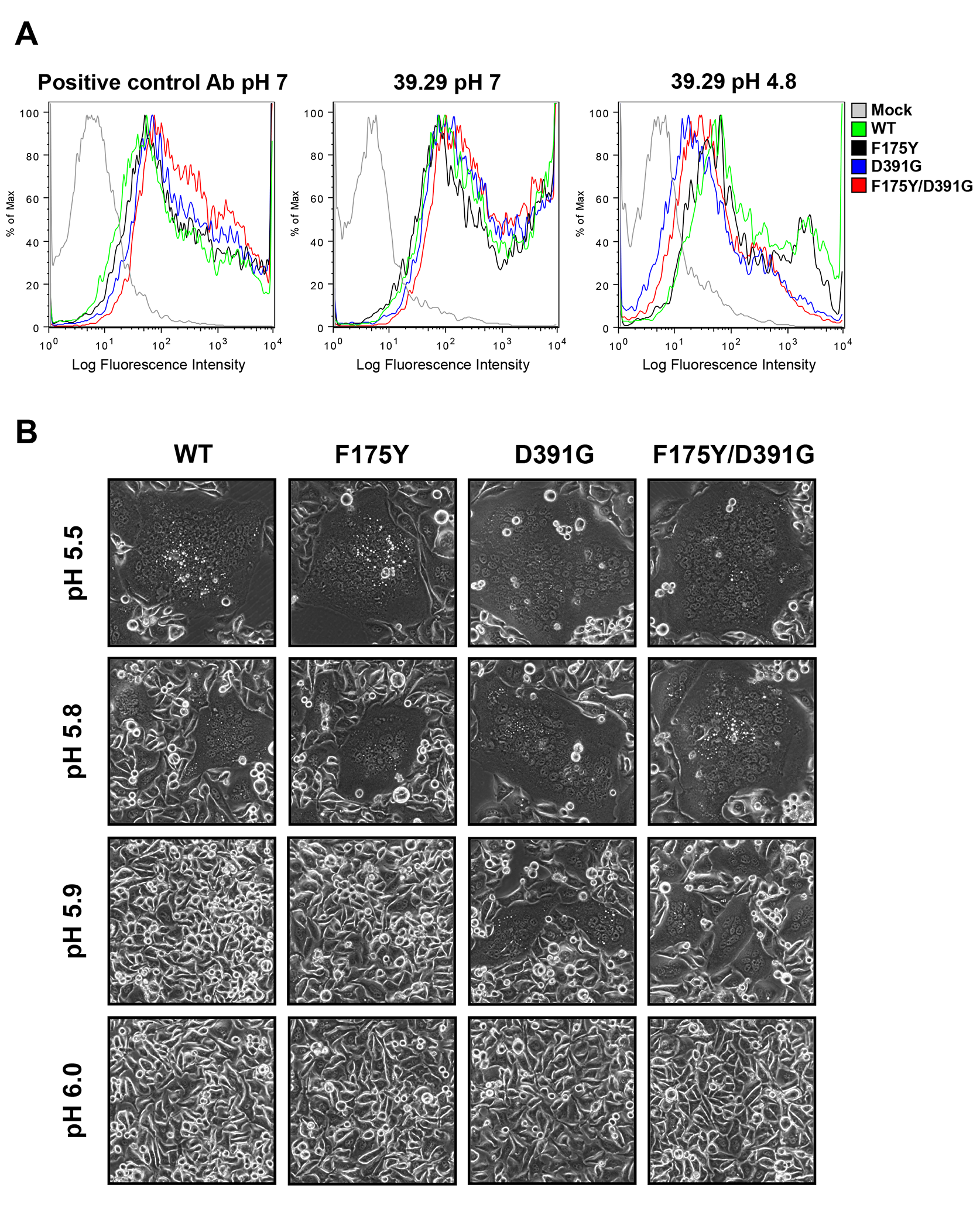

Supplement: S1 Fig — (A) 293T cells expressing the WT, F175Y, D391G or F175Y/D391G A/Perth/16/2009 HA were incubated with a positive control antibody (left panel) or 39.29 (middle and right panels) at pH 7 (left and middle panels) or 4.8 (right panel). Flow cytometry profiles are shown. Mock, mock transfected cells. (B) Hela cells expressing the WT, F175Y, D391G or F175Y/D391G A/Perth/16/2009 HA were treated with trypsin to activate HA0 and then incubated with buffers at different pHs for 2 minutes to induce cell-cell fusion. After overnight culture, representative images were obtained under a phase contrast microscope. (TIF) [file ppat.1005702.s001.tif]

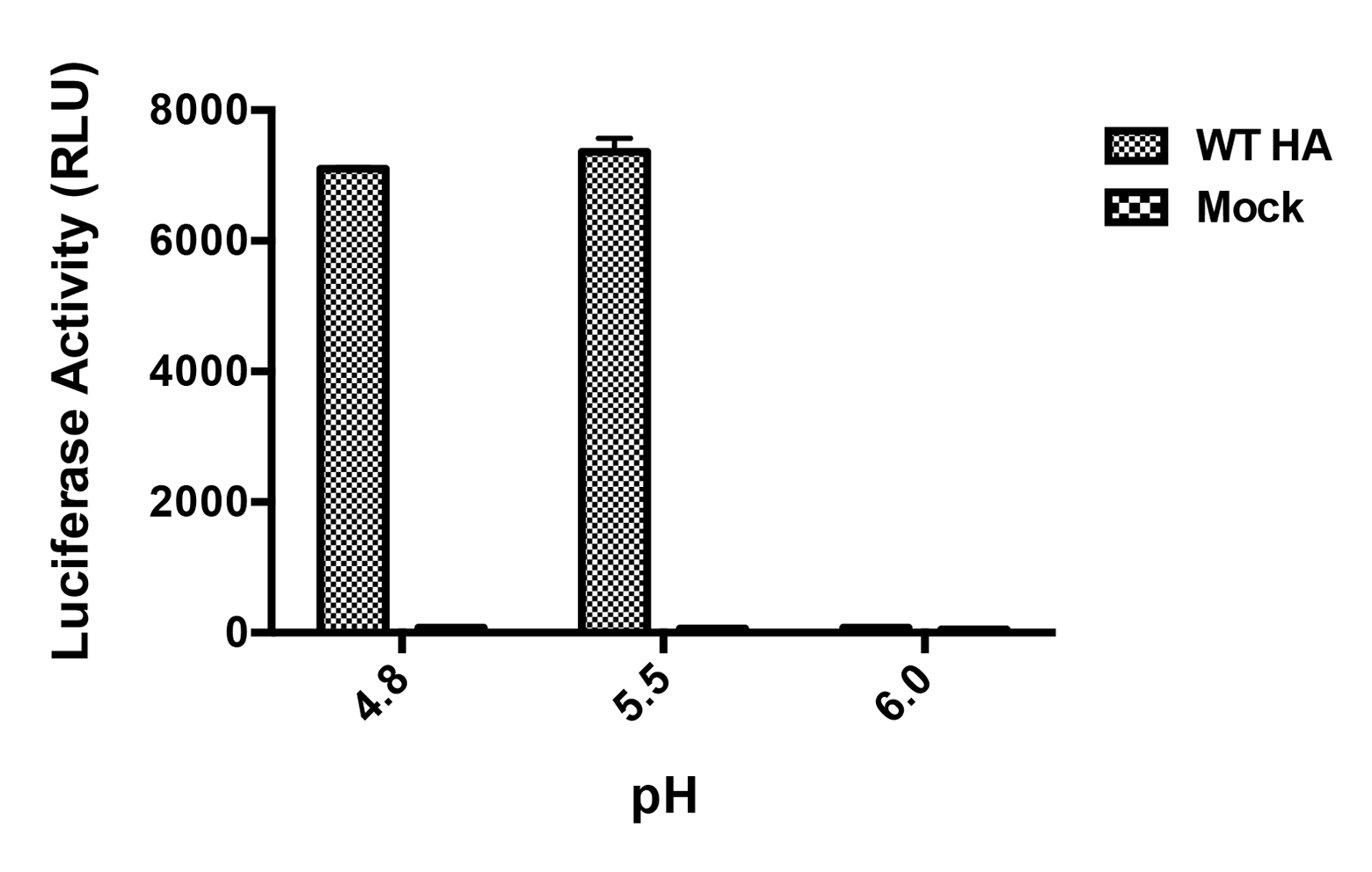

Supplement: S2 Fig — Hela cells expressing the WT A/Perth/16/2009 HA or no HA (transfected with the empty plasmid) plus a tetracycline (Tet)-inducible luciferase protein were mixed with Hela Tet-On 3G cells expressing the WT HA or no HA, respectively. Cells were treated with trypsin to activate HA0 and then incubated with buffers at different pHs for 2 minutes to induce cell-cell fusion. After overnight culture, cells were lysed and incubated with a luminescent substrate of the luciferase. Luminescence signals were measured and are shown as histograms in random luminescence units (RLU). The assay was done in triplicate with data presented as Mean +/- SEM. (TIF) [file ppat.1005702.s002.tif]

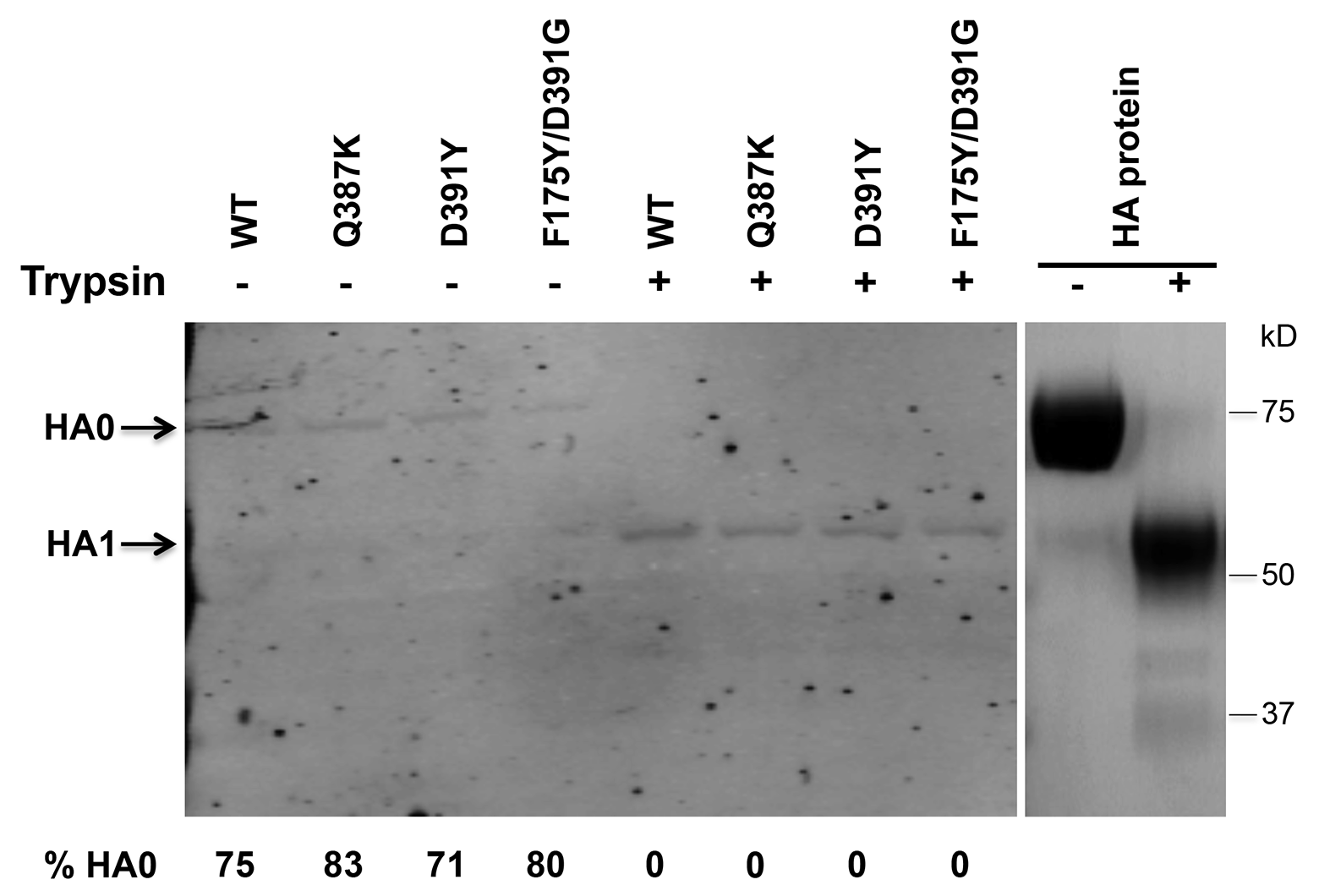

Supplement: S3 Fig — 293T cells expressing the WT A/Perth/16/2009 HA or the Q387K, D391Y or F175Y/D391G mutant HA were collected before and after trypsin treatment. Cells were lysed in Triton Lysis Buffer and the lysates were subjected to SDS-PAGE and Western blot analysis with an antibody against the HA1 subunit of H3 HA. % HA0 is the band intensity of HA0 divided by the band intensities of HA0 + HA1. As a control, a purified HA protein was digested by trypsin and analyzed under the same conditions. (TIF) [file ppat.1005702.s003.tif]

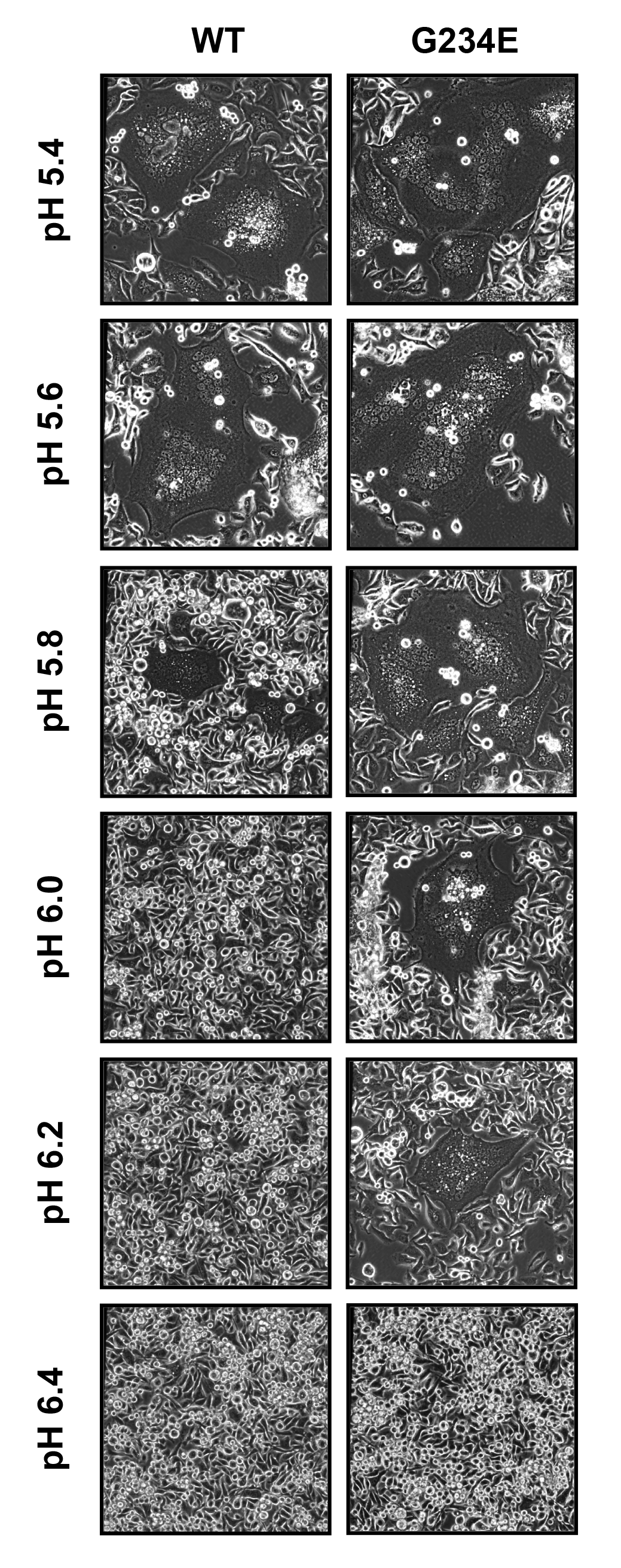

Supplement: S4 Fig — Hela cells expressing the WT or G234E A/Perth/16/2009 HA were treated with trypsin to activate HA0 and then incubated with buffers at different pHs for 2 minutes to induce cell-cell fusion. After overnight culture, representative images were obtained under a phase contrast microscope. (TIF) [file ppat.1005702.s004.tif]

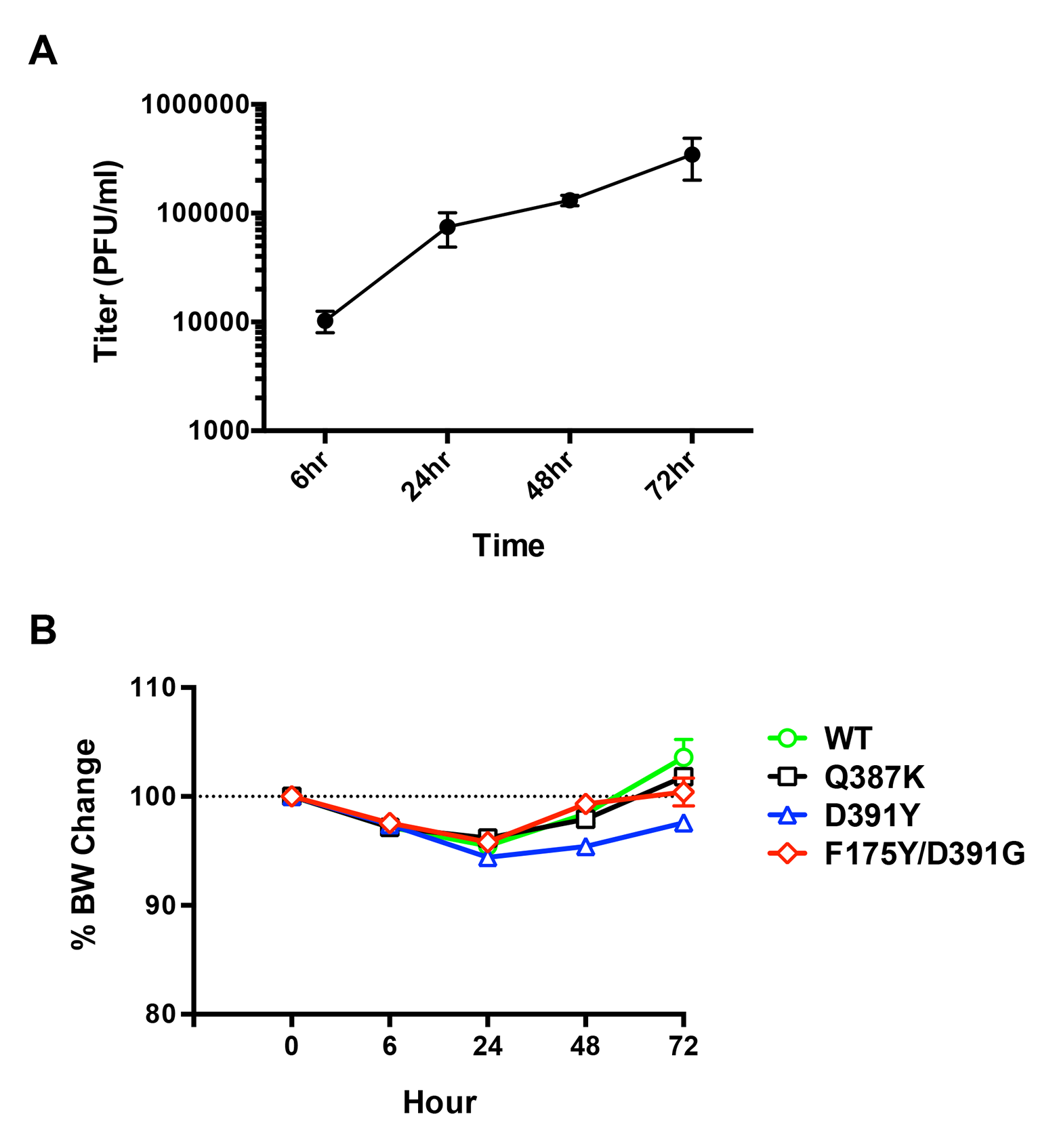

Supplement: S5 Fig — (A) DBA/2J mice were infected with WT A/Perth/16/2009 intranasally. At 6 hr, 24 hr, 48 hr and 72 hr post-infection, lung homogenates were prepared and viral titers in the homogenates were determined on MDCK cells. Each time point contained 5 mice. Lung titers were presented as Mean +/- SEM. (B) DBA/2J mice were infected with same dose of WT or 39.29-resistant A/Perth/16/2009 viruses. At 0 hr (pre-infection), 6 hr, 24 hr, 48 hr and 72 hr post-infection, BW was recorded and plotted as percent BW change compared to pre-infection BW. Each group at each time point contained 5 mice. Data were presented as Mean +/- SEM. (TIF) [file ppat.1005702.s005.tif]

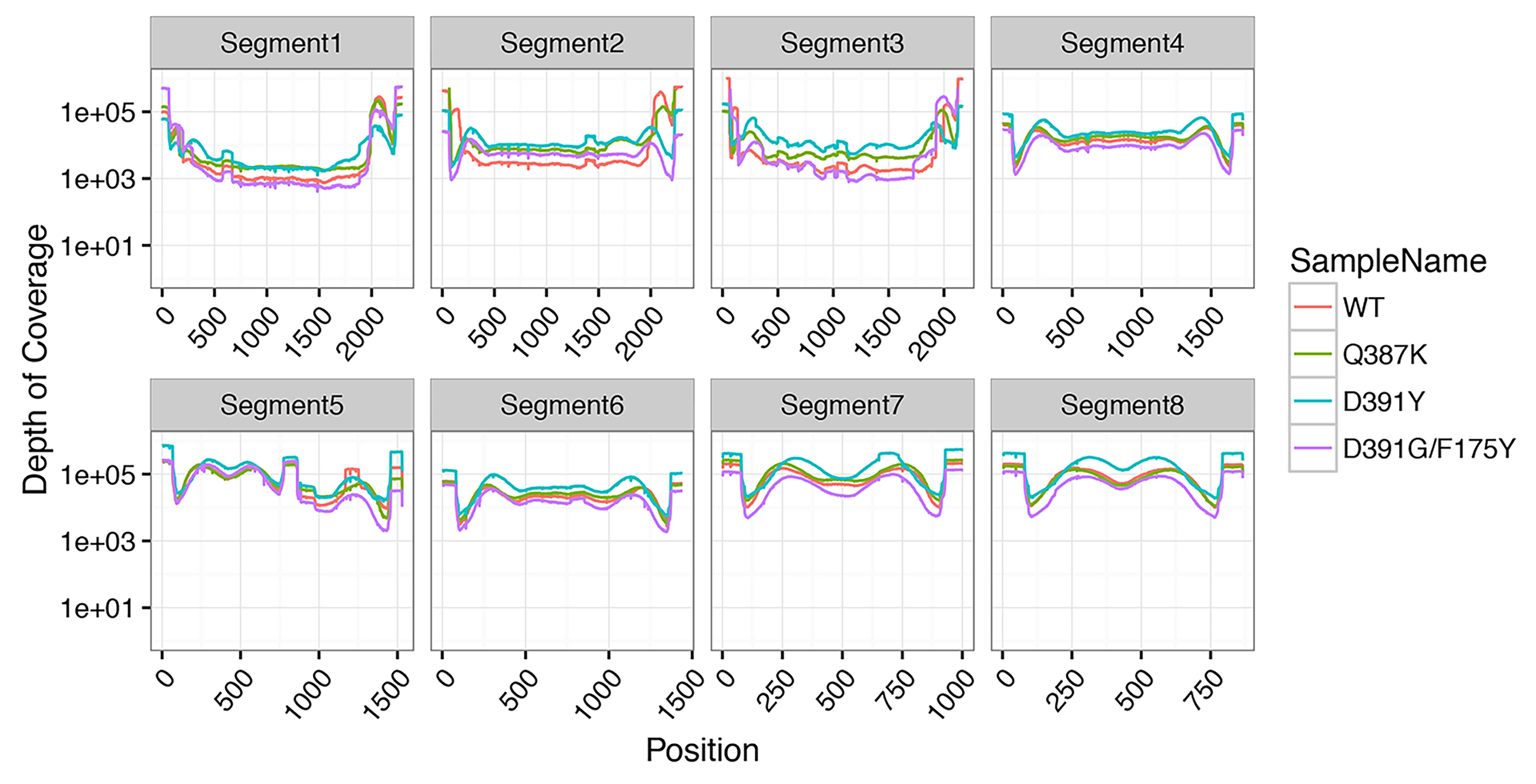

Supplement: S6 Fig — (TIF) [file ppat.1005702.s006.tif]

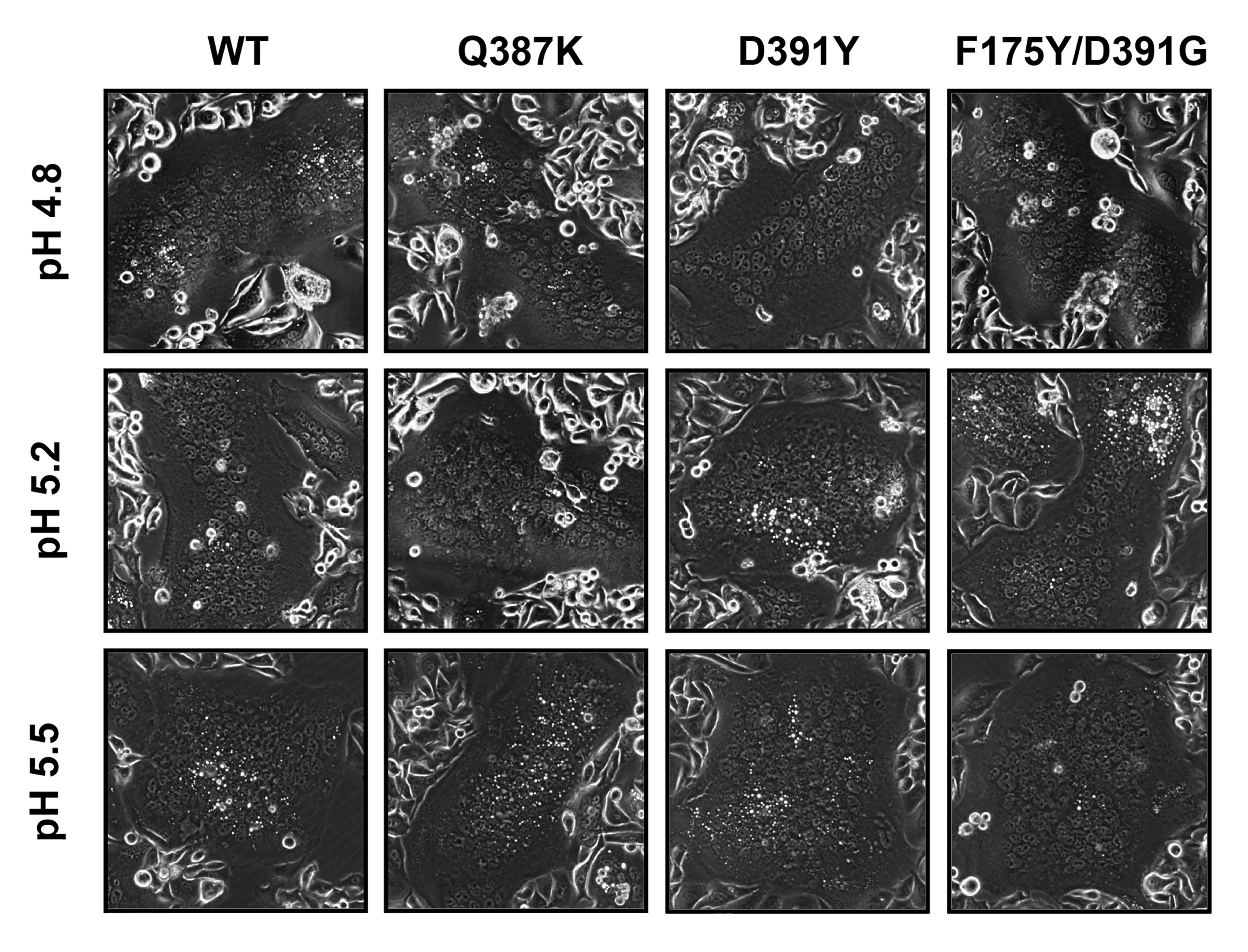

Supplement: S7 Fig — (A) Hela cells expressing the WT or mutant A/Perth/16/2009 HAs were treated with trypsin to activate HA0 and then incubated with buffers at different pHs for 2 minutes to induce cell-cell fusion. After overnight culture, representative images were obtained under a phase contrast microscope. (TIF) [file ppat.1005702.s007.tif]
