# Supplementary material for: Two Escape Mechanisms of Influenza A Virus to a Broadly Neutralizing Stalk-Binding Antibody
Source: PLoS Pathog. 2016 Jun 28;12(6):e1005702. doi: 10.1371/journal.ppat.1005702 (PMC4924800; doi:10.1371/journal.ppat.1005702)
Supplement: S1 Table — (DOCX) [file ppat.1005702.s008.docx]

**S1 Table. Genomic coordinates for each gene in the A/Perth/16/2009 reference sequences (GenBank Accession No. KJ609203 - KJ609210).**

| **Segment** | **Start** | **End** | **Width** | **Strand** | **Gene** | **Exon** |
| --- | --- | --- | --- | --- | --- | --- |
| **Segment1** | 3 | 2282 | 2280 | + | polymerase PB2 | 1 |
| **Segment2** | 1 | 2274 | 2274 | + | polymerase PB1 | 1 |
| **Segment2** | 95 | 367 | 273 | + | PB1-F2 protein | 1 |
| **Segment2** | 118 | 2274 | 2157 | + | PB1-N40 protein(718) | 1 |
| **Segment3** | 5 | 574 | 570 | + | PA-X protein | 1 |
| **Segment3** | 576 | 764 | 189 | + | PA-X protein | 2 |
| **Segment3** | 5 | 2155 | 2151 | + | polymerase PA | 1 |
| **Segment3** | 467 | 2155 | 1689 | + | Unknown | 1 |
| **Segment4** | 9 | 1709 | 1701 | + | hemagglutinin | 1 |
| **Segment5** | 24 | 1520 | 1497 | + | nucleocapsid protein | 1 |
| **Segment6** | 13 | 1422 | 1410 | + | neuraminidase | 1 |
| **Segment7** | 15 | 773 | 759 | + | matrix protein 1 | 1 |
| **Segment7** | 15 | 40 | 26 | + | matrix protein 2 | 1 |
| **Segment7** | 729 | 996 | 268 | + | matrix protein 2 | 2 |
| **Segment8** | 17 | 709 | 693 | + | nonstructural protein 1 | 1 |
| **Segment8** | 17 | 46 | 30 | + | nuclear export protein | 1 |
| **Segment8** | 519 | 854 | 336 | + | nuclear export protein | 2 |
